# Examining the Effectiveness of Neuropsychological Interventions on Cognition in Patients with Epilepsy: A Systematic Review and Meta-Analysis

**DOI:** 10.1007/s11065-025-09678-2

**Published:** 2025-10-23

**Authors:** Irene Cano-López, Judit Catalán-Aguilar, Esperanza González-Bono

**Affiliations:** Institut d’Investigació en Psicologia Dels Recursos Humans, del Desenvolupament Organitzacional i de La Qualitat de Vida Laboral (IDOCAL)/Department of Psychobiology, Psychology Center, Universitat de València, Valencia, Spain

**Keywords:** Neuropsychological intervention, Epilepsy, Cognition, Cognitive rehabilitation

## Abstract

This meta-analysis evaluated the effectiveness of neuropsychological interventions in enhancing cognition in patients with epilepsy. The systematic review was conducted under PRISMA guidelines. Of 1363 articles, 25 met the inclusion criteria. Meta-analyses assessing pre-post changes in experimental interventions (i.e., without comparison to control groups) revealed moderate effects on cognition in adults (*g* = 0.29; 95% CI = 0.18, 0.40; *p* < 0.0001), with significant effects for attention (*g* = 0.24; 95% CI = 0.06, 0.43; *p* = 0.0098), immediate memory (*g* = 0.34; 95% CI = 0.21, 0.47; *p* < 0.0001), delayed memory (*g* = 0.38; 95% CI = 0.18, 0.57; *p* < 0.0001), and language (*g* = 0.33; 95% CI = 0.06, 0.59; *p* = 0.0159). In pediatric samples, moderate effects were found on overall cognition (*g* = 0.55; 95% CI = 0.22, 0.89; *p* = 0.0012), with gains in attention (*g* = 0.66; 95% CI = 0.15, 1.17; *p* = 0.01) and working memory (*g* = 0.80; 95% CI = −0.05, 1.65; *p* = 0.06). Comparisons with control groups (i.e., patients without intervention) showed a trend towards positive effects in adults (*g* = 0.35; 95% CI = −0.00, 0.71; *p* = 0.053), with brain training games associated with poorer outcomes (*B* = −1.03; SE = 0.52; 95% CI = −2.05, −0.00; *p* = 0.049). No significant differences were found in pediatric samples (*g* = 0.34; 95% CI = −0.22, 0.89; *p* = 0.24). The findings support the implementation of targeted cognitive interventions in clinical practice, offering evidence-based recommendations.

**Supplementary Information:**

The online version contains supplementary material available at https://doi.org/10.1007/s11065-025-09678-2.

## Introduction

Epilepsy is one of the most prevalent neurological diseases, affecting approximately 70 million people worldwide (Thijs et al., 2019). It is characterized by a predisposition to seizures, along with neurobiological, cognitive, psychological, and social consequences (Fisher et al., 2014). Specifically, patients with epilepsy have variable patterns of cognitive deficits (Helmstaedter & Kockelmann, 2006) , with memory complaints being among their main concerns (Thompson et al., 2016), as well as frequent emotional disturbances (Pham et al., 2017) that may impact their quality of life (Cano-López et al., 2023). The neuropsychological profile of patients with epilepsy may be influenced by modulator factors such as age (Cano-López et al., 2017); epilepsy type (Arrotta et al., 2022; Cano-López et al., 2025); side of seizure focus (Cano-López et al., 2017; Phuong et al., 2021); seizure frequency and type (Cano-López et al., 2017); age at epilepsy onset (DeGeorge et al., 2021); and treatment characteristics (DeGeorge et al., 2021; Lozano-García et al., 2024a).

Neuropsychological intervention has emerged as a promising approach to address cognitive deficits in patients with epilepsy due to its potential to promote neuroplasticity in other populations with brain injury (Wilson et al., 2015). This intervention includes two main strategies: compensatory and restorative. Compensatory approaches typically focus on helping individuals manage cognitive deficits, minimizing their impact through internal strategies such as visual imagery training (e.g., face-name associations), verbal-based learning (e.g., acronyms), and spaced retrieval training, and the use of external aids (such as diaries, calendars, notebooks, and mobile phones) (Wilson et al., 2009). In contrast, restorative approaches focus on enhancing specific cognitive processes through repeated practice of tasks that have a high load in the cognitive domain of interest, without providing explicit strategy instruction (Lustig et al., 2009). This restorative approach constitutes the basis of what is popularly termed “brain training.” Brain training typically involves repeated practice of cognitively demanding tasks targeting specific domains, often through interactive games on digital devices that allow for adjusting difficulty levels depending on the individual’s performance, provide real-time feedback, and monitor performance over time (Nguyen et al., 2022).

Despite the potential cognitive benefits of neuropsychological intervention in patients with epilepsy, its effectiveness is still underexplored. To date, no meta-analysis has assessed this issue, although some systematic reviews have offered preliminary insights into its potential impact. Farina et al. (2015), in a systematic review that included nine experimental studies, suggested that cognitive rehabilitation could be a valuable approach for improving cognitive deficits in patients with epilepsy. However, they emphasized the need for further controlled studies with well-defined patient groups to clarify its effectiveness and assess the influence of intervention type and duration. Mazur-Mosiewicz et al. (2015), in a systematic review of four studies focused exclusively on cognitive rehabilitation after epilepsy surgery, highlighted that these interventions are underreported and emphasized the need for a systematic evaluation of their effectiveness. Joplin et al. (2018) specifically focused on memory rehabilitation in epilepsy by reviewing 12 studies and concluded that the available evidence was generally weak and inconsistent. While their findings provided preliminary support for verbal memory improvements in adults, evidence for its effectiveness in children is inconclusive.

Therefore, this study aims to evaluate the effectiveness of neuropsychological interventions in enhancing cognitive functioning in patients with epilepsy through a systematic review and meta-analysis. To the best of our knowledge, this is the first meta-analysis to address this topic, and it also examines potential factors that may modulate this effectiveness, including epilepsy treatment-related factors, the characteristics of neuropsychological intervention, and the level of methodological quality of the studies. Considering that adult and pediatric populations significantly differ in terms of neurodevelopment, clinical presentation, and type of intervention – as epilepsy in children evolves alongside central nervous system maturation (Marks et al., 2003) –, separate meta-analyses will be performed for studies including adult and pediatric samples.

## Methods

### Search Strategy and Information Sources

The systematic review was conducted following the Preferred Reporting Items for Systematic Reviews and Meta-Analyses (PRISMA) recommendations (Page et al., 2021) and was registered in PROSPERO (CRD42024625503).

A search was conducted in Web of Science, PsycINFO, Pubmed/MEDLINE, Scopus, and Embase, from inception to November 25, 2024. The search strategy was formulated as follows: (epilep* OR seizure) AND ((“neuropsychological intervention” OR “cognitive intervention”) OR (“neuropsychological rehabilitation” OR “cognitive rehabilitation”) OR (“neuropsychological stimulation” OR “cognitive stimulation”) OR (“neuropsychological training” OR “cognitive training”) OR (“neuropsychological remediation” OR “cognitive remediation”) OR (“neuropsychological strategies” OR “cognitive strategies”) OR (“compensat*” OR “cognitive compensat*”) OR (“cognitive aid” OR “mnemonic aid”)) AND (cognit* OR memory OR attention* OR “executive function*” OR language OR naming OR “verbal fluency”). These keywords were examined in the title section, keywords, and abstract. Furthermore, a reverse search was conducted by reviewing the reference lists of the studies to find articles not included in the databases. The search was not limited by date.

### Eligibility Criteria

The inclusion criteria were as follows: (a) participants with a diagnosis of epilepsy; (b) studies that included a neuropsychological intervention consisting of tasks focused on the improvement of cognitive functions; (c) studies that assessed change in objective measures of cognitive functioning (such as attention, executive functions, memory, and language) after intervention; and (d) original and empirical studies written in English or Spanish, the two languages in which the review team had proficiency, thereby ensuring accurate screening and data extraction. This included both peer-reviewed journal articles as well as non-peer-reviewed sources such as conference proceedings, dissertations, study reports, book chapters, and other forms of grey literature, regardless of publication status, as recommended (Borenstein et al., 2009; Giustini, 2019). We excluded the following: (a) studies with mixed data on people with epilepsy and other patient groups that did not present data separately for the group of patients with epilepsy, (b) studies exclusively including people with psychogenic non-epileptic seizures, (c) animal studies, and (d) reviews and meta-analyses. Studies were not excluded due to age (such as children or adults), the etiology of epilepsy, the type of epilepsy, the treatment (i.e., surgery), or the inclusion of a control group. Although randomized controlled trials offer the highest level of evidence, non-controlled studies were also included to capture the full scope of available empirical research, given the limited number of controlled trials in this population.

### Selection Process

Two authors (ICL and JCA) independently reviewed the titles and abstracts to decide their possible agreement with the inclusion criteria using Rayyan software (Ouzzani et al., 2016). Each researcher then individually assessed the full text of the selected studies to determine whether they met the inclusion criteria. Discrepancies were resolved through critical discussion with a third reviewer (EGB) until 100% agreement was reached.

### Data Extraction

Two authors (ICL and JCA) independently extracted data from all the included studies as follows: (a) reference; (b) sample size, age, and gender; (c) epilepsy type; (d) epilepsy treatment; (e) characteristics and number of sessions of the experimental intervention; (f) characteristics and number of sessions of the control intervention (if any); (g) assessment time; (h) neuropsychological assessment instruments; and (i) results of the intervention in terms of cognitive functioning. These data were verified by a third reviewer (EGB), and discrepancies were resolved through discussion until 100% agreement was reached.

### Data Synthesis

Data extracted from the included articles were divided into five categories: (a) characteristics of the sample; (b) intervention characteristics; (c) neuropsychological assessment; and (d) effects of the neuropsychological intervention in attention, executive functions, memory, and language.

### Methodological Quality Assessment

The methodological quality of the studies was evaluated independently by two reviewers (JCA and ICL). Any disagreements were addressed through discussion and resolved by consensus. One more reviewer (EGB) was consulted when necessary. The critical appraisal checklist for quasi-experimental studies (Barker et al., 2024) was used to evaluate the risk of bias in all the included studies. It consists of nine items aimed at evaluating key aspects of methodological quality. Furthermore, to assess the strength of evidence of the selected studies, the levels of evidence classification system proposed by Melnyk and Fineout-Overholt (2022) were used. This framework distinguishes seven levels of evidence: level 1 (evidence from a systematic review or meta-analysis of all relevant randomized controlled trials); level 2 (evidence from at least one well-designed randomized controlled trial); level 3 (evidence from a single well-designed controlled trial without randomization, a systematic integrative review encompassing a full range of evidence levels, or mixed methods intervention studies); level 4 (evidence from well-designed case-control or cohort studies); level 5 (evidence from systematic reviews of descriptive and qualitative studies); level 6 (evidence from a single descriptive or qualitative study, evidence-based practice, evidence-based quality improvement, or quality improvement projects); and level 7 (evidence from the opinion of authorities and/or reports of expert committees, as well as narrative and literature reviews).

### Meta-Analyses

Multivariate meta-analyses were conducted using the metafor package (Viechtbauer, 2010) within the R software environment (R Core Team, 2021) to account for the statistical dependency among multiple effect sizes reported within the same study (Cheung, 2019). We carried out a main multivariate meta-analysis to evaluate the pre-post changes in experimental interventions across all studies. For studies that included a control group of epilepsy patients who did not undergo neuropsychological intervention (e.g., waiting list), we conducted a multivariate meta-analysis to compare the pre-post changes between experimental and control groups. A hierarchy of assessment instruments was established for each cognitive domain based on their frequency of use in the studies included in the meta-analysis. Data entry prioritized the highest-ranked instrument for each study. When studies reported data from multiple follow-up periods, the meta-analysis included only the data from the immediate post-intervention period. When two or more experimental interventions were administered in the studies, intervention groups were combined in a single group, and the weighted mean and SD were obtained. When mean and SDs were not available, the corresponding authors of the included studies were contacted to request data as recommended (Higgins et al., 2024). If the corresponding author did not respond or was unable to provide the requested data, the article was excluded from the meta-analyses. Considering the relevance of the differences between child and adult populations in terms of neurodevelopment, clinical presentation of epilepsy, and type of intervention (Marks et al., 2003), we performed separate multivariate meta-analyses for adults and children.

We employed a multivariate meta-analysis of multiple endpoint studies, with effect sizes nested within studies by cognitive outcome (i.e., attention, executive function, memory, and language), allowing us to retain multiple outcomes per study while appropriately modeling their statistical dependence. The models specified an unstructured between-study covariance matrix, thereby estimating both the between-study variances for each outcome and the covariances between outcomes. Sampling variances and covariances were represented through block-diagonal matrices, assuming a constant correlation of 0.5 between effect sizes within the same study (Cheung, 2019). To assess the robustness of this assumption, sensitivity analyses were performed using alternative correlation values (0 and 0.8) (Cheung, 2019). The meta-analysis was implemented for continuous data using inverse variance weights with a 95% confidence interval. We specifically selected a random-effects model because our goal was to generalize the findings beyond the included studies, assuming they represent a random sample from a broader population of relevant studies. This approach accounts for both sampling error and heterogeneity in effect sizes arising from differences in intervention type, duration, outcome measures, and other study-level characteristics (Borenstein et al., 2010). The between-study variance was estimated using the restricted maximum likelihood (REML) estimator. Effect sizes were calculated using Hedges’ (adjusted) *g* effect size (standardized mean difference). For meta-analyses examining the pre-post changes in experimental interventions, Hedges’ *g* was computed as the mean difference between post- and pre-intervention scores divided by the standard deviation of the pretest scores (Morris & DeShon, 2002), with an estimated correlation of 0.70 between the pre- and post-intervention measures of the same outcome from the same study (Rosenthal, 1991). In the case of meta-analyses comparing the pre-post changes between experimental interventions and control groups of patients with epilepsy without neuropsychological intervention, effect sizes were computed as the difference between the standardized mean changes of the experimental and control interventions, divided by a pooled estimate of the pretest standard deviation (Morris, 2008). The full formulas used to compute both Hedges’ *g* and its sampling variance are provided in the supplementary material. The values used to interpret Hedges’ *g* estimates followed established guidelines, with a small effect size defined as 0.20 or lower (range 0–0.20); moderate = 0.50 (range 0.30–0.70); and large = 0.80 (≥ 0.80) (Cohen, 1988; Durlack, 2009). We computed 95% prediction intervals to estimate the expected range of true effects, using the recommended formula (Borenstein, 2024) (Supplementary material). Prediction intervals were calculated separately for each outcome using its outcome-specific *τ*^2^ estimate. Furthermore, a single global prediction interval based on a pooled *τ*^2^ was reported, weighted by the number of studies contributing to each outcome.

The presence of outliers and influential cases was examined using studentized deleted residuals (*z*), Cook’s distances, and DFBETAs for each study, implemented in the metafor package, following the recommendations of Viechtbauer and Cheung (2010). Studies with absolute studentized deleted residuals greater than 1.96 were identified as potential outliers and considered for further inspection, as such values suggest the study’s effect size deviates more than expected under the assumed model (Viechtbauer & Cheung, 2010). Cook’s distance estimates how much a study influences the model, with high values indicating that its removal would substantially alter the results (Cook & Weisberg, 1982). Finally, DFBETAs examines the change in the overall effect size estimate from a random-effects model when excluding each study, with values above 1 being considered to indicate influential cases (Viechtbauer & Cheung, 2010). The identification of potential outliers was not used as a sole criterion for exclusion, but rather as an indication for further inspection of study-level characteristics and methodological consistency.

To examine the presence of small-study effects, we first applied Egger’s regression test (Egger et al., 1997), adapted to multivariate meta-analyses by modeling effect sizes as a function of their precision (1/√variance) using an unstructured variance-covariance matrix. Funnel plot asymmetry identified by this test indicates that smaller studies systematically differ from larger ones, a phenomenon often consistent with publication bias but also potentially attributable to other sources such as poor methodological design of small studies or true heterogeneity in effects (Egger et al., 1997). When evidence of potential publication bias was detected, we estimated a bias-adjusted overall effect size using selection models. Since these models are not currently implemented for multivariate meta-analyses in the metafor package, we followed the strategy recommended by Nakagawa et al. (2022), which involves aggregating multiple effect sizes within studies to ensure statistical independence. Specifically, effect sizes were aggregated using inverse-variance weighting, assuming an intra-study correlation of 0.5. A three-parameter selection model (Vevea & Hedges, 1995), which accounts for publication bias by estimating the probability of study inclusion based on statistical significance, was then fitted to the aggregated data using the selmodel() function from metafor, with a *p*-value threshold set at 0.05 (Nakagawa et al., 2022).

To assess whether the effectiveness of the interventions varied across cognitive domains, we first conducted a multivariate meta-analysis including only the cognitive outcome as a categorical moderator. This initial model allowed us to estimate domain-specific effect sizes and evaluate potential differences in intervention efficacy by cognitive domain. Subsequently, we performed a multivariate meta-regression to examine whether intervention effects were influenced by both cognitive outcome domain and additional study-level characteristics. The final model included the type of cognitive outcome, epilepsy treatment (surgery or not surgery), use of restorative strategies involving brain training games (i.e., yes or no), use of compensatory strategies (internal strategies only, combined internal and external strategies, or none), intervention format (individual vs. group-based), intervention duration, and methodological quality (using the level of evidence classification as an ordinal moderator, with higher values indicating lower quality).

## Results

### Study Selection

**Fig. 1:**
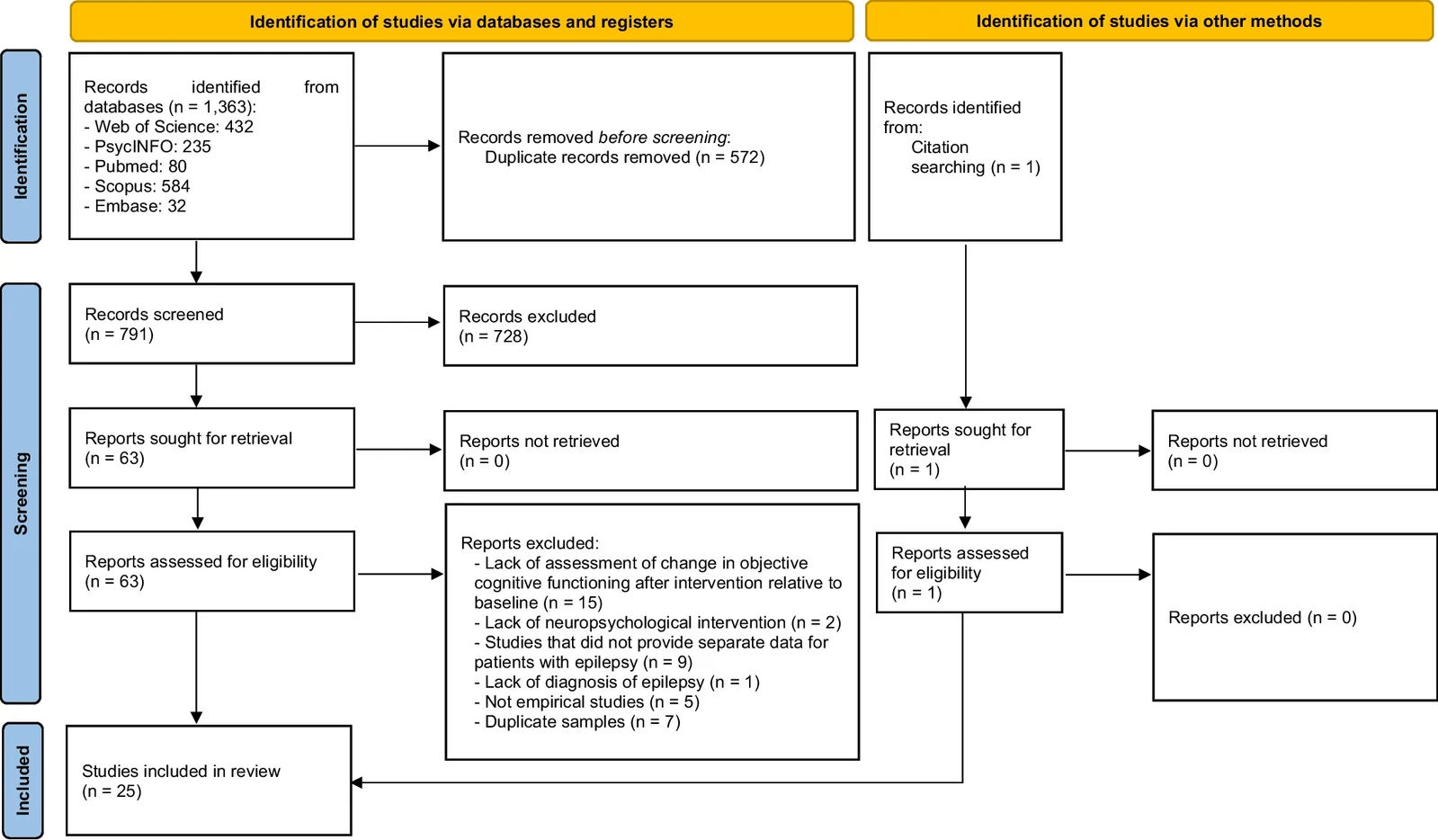
Flow diagram

The initial search resulted in a total of 1,363 articles. After removing duplicates, the titles and abstracts of 791 articles were screened, and 728 studies were excluded for not meeting the inclusion criteria. Consequently, the full text of 63 articles was reviewed. Of these, 39 were excluded for the following reasons: lack of assessment of change in objective cognitive functioning after intervention relative to baseline (*n* = 15), lack of neuropsychological intervention (*n* = 2), not providing separate data for patients with epilepsy (*n* = 9), lack of diagnosis of epilepsy (*n* = 1), no empirical studies (*n* = 5), or duplicate samples (*n* = 7). According to the Cochrane Handbook for Systematic Reviews of Interventions, duplicate publications can introduce substantial bias into meta-analyses (Higgins & Green, 2011). In our review, the article by Khaleghi et al. (2023), published in Arabic, involved the same sample as the later article by Khaleghi et al. (2024), published in English; therefore, only the latter was included to avoid double counting. Similarly, the conference abstracts by Caller et al. (2014, 2016b) and Jobst et al. (2016) reported data from the same sample as the full article by Caller et al. (2016a). The abstracts by Sharma et al. (2021a, b) duplicated data later published in Sharma et al. (2024), and the abstract by Streltzov et al. (2017) overlapped with the sample reported in Streltzov et al. (2022). Since only the full articles by Caller et al. (2016a), Sharma et al. (2024), and Streltzov et al. (2022) provided sufficient data for meta-analytic purposes, they were selected instead of the corresponding conference abstracts. After excluding studies during the full-text review phase, 24 studies were selected, and one study found through reverse searching was also included. In total, 25 studies met the inclusion criteria and were included in the systematic review. Despite efforts to identify and include grey literature, all selected studies were peer-reviewed journal articles. Furthermore, all studies that met the eligibility criteria were published in English. Included studies are denoted with a symbol (*) in the references section. The flow diagram of the study selection process is shown in Fig. 1, and the summary of the selected studies is shown in Tables 1 and 2.

**Table 1:** Summary of studies with adult patients

Reference	Sample size; mean age ± SD; % of women	Epilepsy type	Epilepsy treatment	Experimental intervention		Control intervention		Assessment time	Neuropsychological assessment instruments	Results
				Characteristics of intervention	Number of sessions and duration	Characteristics of intervention	Number of sessions and duration			
Anuradha et al. (2014)	EG: *n *= 15 PWE; 31.33 ± 6.67 years; 60% women CG: *n *= 15 PWE; 30.20 ± 6.83 years; 40% women	n.s	ASMs	Tasks for general cognitive improvement (e.g., financial planning, and simple maths) and tasks for specific cognitive functions (e.g., attention, categorization, and memory) Strategies: internal compensatory Use of brain training games: no Intervention format: individual	30 sessions; total: 30 h	CG: no intervention	-	EG: baseline and immediately post-intervention CG: baseline and after 30 days	- Digit Vigilance Test - DSST - N back test - Tower of London Test - WCST -Immediate and delayed memory (verbal and visual jointly) (instrument: n.s.) -Category fluency	*Baseline:* - n.s.d. between EG and CG *After intervention:* *EG:* - ↑ in attention, verbal and visual memory, mental speed, executive functioning, and response inhibition *CG:* - ↑ in attention, mental speed, memory, direct recall, and semantic fluency
Caller et al. (2016a)	EG1 + EG2: *n *= 29 PWE; 49.3 ± 9.2 years; 66% women EG1: *n *= 15 PWE; age and gender: n.s EG2: *n *= 14 PWE; age and gender: n.s CG: *n *= 20 PWE; 41.4 ± 11.2 years; 65% women	EG1 + EG2: Genetic generalized epilepsy: 14% CG: Genetic generalized epilepsy: 10%	ASMs Surgery: EG1 + EG2: 41% CG: 35%	EG1 + EG2: HOBSCOTCH by a trained coach: patient education, self-monitoring training, problem-solving therapy, and memory strategies following the Memory and Attention Adaptation Training Session 1 and 8: in person; rest of the sessions: by telephone EG2 also performed additional cognitive training with working memory exercises using a Nintendo DS® console and the Brain Age© program Strategies: internal and external compensatory + restorative (EG2) Use of brain training games: yes (EG2) Intervention format: mainly individual (although sessions 1 and 8 in groups)	EG1 + EG2: 8 sessions of 45 to 60 min over 8 weeks; total: 6–8 h (mean 7 h) EG2 also had cognitive training five days a week for 20–40 min, over the 8 weeks of the program; total: 13.3–26.7 h (mean: 20 h) Mean duration (EG1 and EG2): 17 h	CG: no intervention (waiting list)	-	Baseline (T0) and after the intervention (T1)	RBANS	*Baseline*: *EG1* + *EG2 vs CG:* - n.s.d. in memory, visuospatial/constructional, language, and attention *After intervention*: *EG1* + *EG2 vs CG:* - Better performance in attention and scores in the RBANS total scale *EG1 vs EG2:* - n.s.d. in cognitive performance *EG1* + *EG2:* - ↑ in cognitive performance *CG:* - ↓ in visuospatial memory, language, and attention
de Vasconcelos et al. (2017)	EG: *n *= 9 PWE; 44.0 ± 14 years; 55.6% women CG: *n *= 9 PWE; 41.0 ± 14.4 years; 55.6% women	Left TLE	Surgery ASMs	Psychoeducation and rehabilitation techniques focusing on attention, memory, and word-finding problems: - Cognitive strategies: self-monitoring and encoding level intervention (i.e., semantic elaboration, prospective memory training, and visual imagery) - External aids (e.g., notes and diaries) - Homework was checked in the next session Strategies: internal and external compensatory Use of brain training games: no Intervention format: individual	8 individual sessions of 1 h., once a week for two months; total: 8 h	CG: no intervention	-	EG: - Cognitive assessment: baseline and 30 days after the intervention - fMRI: baseline (T0), 30 days after intervention (T1), and three months after intervention (T2) CG: - Cognitive assessment: baseline and two months later - fMRI: baseline and two months later	- BNT - RAVLT - Ecological Activity of the Neuropsychological Assessment Battery - fMRI study with a fluency paradigm to assess language networks pre- and post-rehabilitation	*Baseline* *EG vs CG:* - n.s.d. in cognitive performance - n.s.d. in activation patterns during the fMRI *After intervention:* *EG* vs CG *based on RCIs:* - 3 patients in the EG improved in verbal learning *vs* 1 in the CG - 2 patients in the EG improved in naming *vs* 1 in the CG *EG:* - ↑ in verbal learning, naming, and ecological and subjective memory - n.s.d. in activation patterns between the first and the second scan - From T0 to T2: differences in the fMRI in the right anterior prefrontal cortex, pars opercularis of Broca’s area in the left hemisphere, and in the right inferior prefrontal gyrus - From T1 to T2: differences in the fMRI in the right dorsal anterior cingulate cortex and the left angular gyrus *CG:* - ↑ in ecological memory - From T0 to T1: differences in the fMRI in the primary somatosensory cortex and the left fusiform gyrus
Gaston et al. (2019)	*N *= 9 PWE with memory complaints; 40.8 ± 12.3 years; 55.6% women	Genetic generalized epilepsy: 2 Left TLE: 1 Right TLE: 1 Bilateral TLE: 3 Right FLE: 1 Unclear seizure onset localization: 1	ASMs	Cognitive rehabilitation program based on the CogSMART and the “Making the Most of Your Memory: an Everyday Memory Skills Program”: - Educational component on memory and/or epilepsy - Education on an internal and external memory strategy with active practice in session - Education on a lifestyle issue pertinent to epilepsy and cognition (e.g., sleep). Strategies: internal and external compensatory Use of brain training games: no Intervention format: group-based	6 sessions of 1.5–2 h, one per week; total: 9–12 h (mean: 10.50 h)	Not included	Not included	- Cognitive assessment: baseline and one week after the intervention - fMRI (only in five patients): before and after the intervention	- RAVLT - BVMT-R - Digit Span subtest of WAIS-IV - fMRI with a verbal learning task with a “read” condition, in which participants were shown a word pair and instructed to read the second word aloud; and a “generate” condition in which participants were given the first word and the first letter of the second word, followed by asterisks representing missing letters, and were instructed to generate the second word aloud	*After intervention:* - ↑ in verbal immediate memory and visuospatial delayed memory - ↑ in the read condition (fMRI) - n.s.d. in working memory, delayed verbal memory, and the generate condition (fMRI) *fMRI:* *Generate condition:* - ↑ spatial activation in left inferior frontal gyrus and bilateral anterior cingulate gyrus - ↓ activation in left superior temporal gyrus and left cerebellar tonsil *Read condition:* - ↑ activation in left cuneus and left inferior frontal gyrus - ↓ activation of right cingulate gyrus, right middle temporal gyrus, right supramarginal gyrus, and ↑ activation in left cuneus
Grewe et al. (2013)	EG: *n *= 5 PWE; 35.04 ± 8.08 years; 20% women CG: *n *= 19 healthy university students; 23 ± 3.45 years; 73.7% women	Focal epilepsy	ASMs	EG: Phase 1 (days 1–6): memorization of an auditorily presented shopping list (list A) to buy them later in the 360° VR supermarket - Phase 2 (day 7): interference phase in which a new shopping list (list B) for memorizing was introduced to buy them later in the 360°- VR supermarket - Phase 3 (day 8): participants had to buy in the 360°-VR supermarket all remembered items of list A again, but without a new presentation of list A Strategies: restorative Use of brain training games: yes Intervention format: individual	Eight sessions; total: 8 h	CG: Phase 1 (days 1–3): memorization of an auditorily presented shopping list (list A) to buy them later in the 360°- VR supermarket - Phase 2 (day 4): interference phase in which a new shopping list (list B) for memorizing was introduced to buy them later in the 360° VR supermarket - Phase 3 (day 5): to buy in the 360°- VR supermarket all remembered items of list A again, but without a new presentation of list A Strategies: restorative Use of brain training games: yes Intervention format: individual	5 sessions; total: 5 h	- Days 1–8 of the intervention (EG) - Days 1–5 (CG)	- Time to buy all the shopping items they remembered - Number of correctly picked items from the respective list - Adjusted number of correctly picked items from the respective list (“product-score”; i.e., number of correct items minus false positives minus repetitions) - Length of movement trajectories	*EG:* - > number of items remembered on day 7 than on day 1 - ↓ in time spent shopping from day 6 to day 8 - n.s.d. in the length of movement paths and the number of correct products between day 6 and day 8 *CG:* - Overall effect of learning the first 3 days for correct products and product-score, as well as in the 5 days of training, including the distraction test *-* ↓ in the length of movement trajectories over the course of the three days of learning, as well as in the five days of training, including the distraction test - ↓ time needed to recall the items over the three days of learning, as well as in the five days of training, including the distraction test
Helmstaedter et al. (2008)	EG: *n *= 55 PWE; 36.2 ± 9.9 years; 45.5% women CG: *n *= 57 PWE; 36.4 ± 9.7 years; 38.6% women	Drug-resistant focal epilepsy 53% hippocampal sclerosis	Temporal lobe surgery EG: 34 selective amygdalohippocampectomy, 21 lesionectomy, 29 right surgery, 26 left surgery CG: 31 selective amygdalohippocampectomy, 26 lesionectomy 26 right surgery, 31 left surgery	Program based on the principles described by Prigatano (2004): - Metacognitive neuropsychological therapy - Cognitive computerized exercises for attention, memory, and executive functions - Occupational therapy - Individual neuropsychological counselling - Sociotherapy Other therapies (such as medical care or physical therapy) were offered individually when indicated The intervention started 3 to 15 days after surgery Strategies: internal and external compensatory + restorative Use of brain training games: no Intervention format: mainly individual, but group-based metacognitive therapy	- Metacognitive neuropsychological group therapy: five sessions of 1 h. each week - Cognitive computerized exercises: four to five sessions per week (on average) - Occupational therapy: three to four sessions per week - Individual counselling two to three times per week Intervention mean duration: 29.3 days; estimated total: 44 h	CG: no intervention	-	Before and three months after temporal lobe surgery	- VLMT - DCS - Letter cancellation test	*Baseline:* *EG vs CG:* - > performance in verbal learning, nonverbal memory, and attention than CG *% of patients with impairment (EG vs CG):* Attention: 29% *vs* 49% Verbal learning: 31% *vs* 59% Delayed free recall: 38% *vs* 58% Figural memory: 25% *vs* 59% *After intervention:* - ‘‘Rehabilitation x Surgery’’ effects on recognition and verbal learning: ↑ or stabilization in the EG and ↓ in the CG - Considering the side of surgery: interaction effect of ‘‘Surgery x Rehabilitation’’ on all memory parameters for right TLE-resected patients, but not for left TLE-resected patients - No effects of rehabilitation on visual memory and attention
Icellioglu et al. (2017)	*N *= 9 PWE; 36.2 ± 10.7 years; 77.8% women	Symptomatic epilepsy (*n *= 6); cryptogenic epilepsy (*n *= 3) TLE (*n *= 7); OLE (*n *= 1); unknown (*n *= 1)	ASMs	Computerized exercises focused on training attention, memory, and executive functions with different levels of difficulty. Sessions supervised by a psychologist Strategies: restorative Use of brain training games: yes Intervention format: individual	One weekly session of 45 min for eight weeks; total: 6 h	Not included	Not included	Baseline and three months after the completion of the intervention	- Stroop test - SRT - SPART - DSST - Verbal Fluency Test	*After intervention:* - ↑ processing speed, spatial delayed recall, and lexical fluency - n.s.d. in verbal learning
Jones (1974)	EG1: *n *= 18 left TLE; 36.2 years, SD: n.s., range 15–59 years; 27.8 women EG2: *n *= 18 right TLE; adults; 27.2 years, SD: n.s., range 16–47 years; 22.2 women EG3: *n *= 2 bilateral mesial temporal damage; adults; 46.0 years, SD: n.s., range 45–47 years; 0% women CG1: *n *= 18 healthy participants; 26.6 years, SD: n.s., range 17–51 years; 22.2% women CG2: *n *= 18 healthy participants; 27.6 years, SD: n.s., range 16–49 years; 22.2% women	Left TLE (*n *= 18); right TLE (*n *= 18); amnesic syndromes (*n *= 2)	Right anterior temporal lobectomy (*n *= 18); left anterior temporal lobectomy (*n *= 19); bilateral temporal lobe excision (*n *= 1)	EG1, EG2, EG3, and CG1: application of imagery mnemonics for the memorization of paired words (including both abstract and concrete words) Strategies: internal compensatory Use of brain training games: no Intervention format: individual	One session; total: 2 h	CG2: no intervention: memorization trials but without instruction for the use of imagery mnemonics	-	EGs and CGs: during the memorization trials	Three lists of paired words, with three trials in each one: - List I (baseline) to assess the baseline ability to memorize paired words - List II (didactic): explanation with examples of the imagery mnemonics - List III (test): implementation of the image mnemonic technique by the patient The presentation of the lists was immediately followed by the first word of each pair, asking the patient to respond with the second word of the pair	*Immediate recall of concrete pairs:* - EG1 < EG2 - EG2 > CG1 *-* ↑ in performance in the EG1, EG2, and CG1 *Immediate recall of abstract pairs:* - EG1 < other groups - n.s.d. between CG1 and CG2 *Delayed recall of concrete pairs:* - EG1 < other groups *-* ↑ in performance with imagery in all groups - > scores in the list II and III in the CG1 than CG2 - > scores in the list II and III in the EG1 *Delayed recall of abstract pairs:* - EG1 < other groups - n.s.d. between CG1 and CG2 EG3 did not improve despite using imagery
Koorenhof et al. (2012)	EG: *n *= 20 PWE; 36.30 ± 10.80 years; 65% women CG: *n *= 22 healthy people; 43.40 ± 12.7 years; 59% women	Left TLE patients who were candidates for surgery	Surgery: 50% of patients underwent neuropsychological intervention before surgery, and 50% after surgery	EG and half of the sample of the CG: memory training (use of external and internal strategies such as mental imagery, method of loci, and the story method) + Lumosity computerized brain training (memory, concentration, mental flexibility, cognitive control, and processing speed) Strategies: internal and external compensatory + restorative Use of brain training games: yes Intervention format: individual	Memory training: three sessions; total: 4 h Lumosity computerized brain training: 6 to 40 sessions for at least 15 min daily; total mean: 5.75 h Mean total duration: 9.75 h	Half of the sample of the CG: memory training (use of external and internal strategies such as mental imagery, method of Loci, and the story method) Strategies: internal and external compensatory Use of brain training games: no Intervention format: individual	Three sessions; total: 4 h	Baseline (T0) and one month after the intervention (T1)	- Story Recall and the List Learning tests of the BMIPB - Brain Performance Index of Lumosity	*Total sample:* - ↑ verbal recall and verbal learning *EG vs CG:* - n.s.d. in cognitive functioning *EG:* - From T0 to T1: no significant changes *Experimental intervention vs control intervention:* - Lower gains in verbal recall - Greater gains in verbal learning *Experimental intervention:* - Number of sessions of computerised brain training related to higher Brain Performance Index of Lumosity, but not to BMIPB
Maialetti et al. (2020)	Total: *N *= 33 PWE; 49.18 ± 13.95 years; 45% women EG: *n *= 10 PWE; age and gender: n.s CG: *n *= 19 PWE; age and gender: n.s 4 patients without neuropsychological assessment	Brain tumor-related epilepsy	ASMs Surgery: 17 patients gross total resection, 10 partial resection, 4 biopsy, and 2 no intervention	EG: multimodal rehabilitation: epileptological follow-up + supportive meeting groups + social assistance + neuropsychological intervention (individual): restoration of weakened cognitive function + compensation through the use of aids: external/internal and active/passive (calendar, agenda, timer) or through the intervention of residual cognitive resources Strategies: internal and external compensatory Use of brain training games: no Intervention format: individual	12 weekly sessions of 1 h; total: 12 h	CG: epileptological follow-up + supportive meeting groups + social assistance	Epileptological follow-up: monthly Supportive meeting groups every 15 days Social assistance as needed	Baseline and immediately post-intervention	-MMSE - Raven Progressive Matrices - Tower of London - TMT A and B - Visual search - Phonemic and semantic fluency - RAVLT - ROCF: copy, immediate, and delayed recall - CDT	*EG vs CG:* - Baseline: < scores in MMSE, RAVLT immediate and delayed recall, and ROCF immediate recall - Post-intervention: > scores in ROCF delayed recall *EG:* - ↑ verbal immediate and delayed recall (RAVLT) *CG:* - No significant post-intervention changes in cognitive function
Maschio et al. (2015)	*N *= 12 PWE; 49.42 ± 13.98 years; 58% women	Brain tumor-related epilepsy	ASMs	Training NeuroPsicologico software: memory, attention, visuospatial functions, language, and reasoning tasks. The duration of the exercise was individualized for task difficulty Strategies: restorative Use of brain training games: yes Intervention format: individual	10 weekly sessions of 1 h each; total: 10 h	Not included	Not included	Baseline (T0), immediately post-intervention (T1), and 6 months follow-up (T2)	- MMSE - TMT A and B - Raven Progressive Matrices - ROCF: delayed recall - CDT - Span forward and backward - Recall of disyllabic words - Rey-Osterreith Word List immediate and long-term recall - Story recall - Phonemic and semantic fluency	*Change from T0 to T1:* - ↑ Span forward, ROCF-recall, story recall, and phonemic fluency *Change from T0 to T2:* - ↑ Span forward, ROCF-recall, story recall, and phonetic fluency
Paiva et al. (2023)	*N *= 27 PWE; 28.81 ± 10.21 years; 63% women	Juvenile myoclonic epilepsy	ASMs	Cognitive training, compensation, and cognitive remediation. 4 modules: planning/ organization, attention, impulsivity, and review Strategies: internal and external compensatory Use of brain training games: no Intervention format: individual	12 sessions once a 60-min week; total: 12 h	Not included	Not included	Baseline (T0) and 3 months after the intervention (T1)	- Digit span forward and backward - Stroop test - TMT A and B - WCST - COWA - CPT - GDT - IGT	*Change from T0 to T1:* - ↑ in TMT A, Stroop test, COWA, CPT, and WCST - IGT: ↓ choices from block A and C, and ↑ choices from block B and D - GDT: ↑ performance (trend, *p* = 0.06)
Radford et al. (2011)	EG1: early training group: *n *= 17 PWE; 39.70 ± 11.2 years; 59% women EG2: late training group: *n *= 14 PWE; 47.7 ± 9.7 years; 36% women	EG1: TLE (*n *= 6), extratemporal (*n *= 5), other (*n *= 6) EG2: TLE (*n *= 9), extratemporal (*n *= 2), other (*n *= 4)	ASMs Surgery: four patients in EG and five patients in CG	EG1 and EG2: psychoeducation about memory and training in the use of cognitive strategies (internal and external). Group exercises and discussion + homework tasks Strategies: internal and external compensatory Use of brain training games: no Intervention format: group-based	Six weekly 2-h sessions; total: 12 h	Not included	Not included	Waitlist crossover design with 3 assessments, conducted at 12-week intervals EG1: baseline assessment (T0) and 2 post-intervention assessments (T1 and T2) EG2: 2 baseline assessments (T0 and T1) and 1 post-intervention assessment (T2)	- RAVLT - RPA-ProMem - Appointment memory	*Total sample:* - ↑ in RAVLT (total learning and delayed recall) - No changes in RPA-ProMem or appointment memory *EG1 vs EG2:* - From T0 to T1: > improvement in RAVLT total learning score (trend, *p* = 0.06) and delayed recall and appointment memory, n.s.d. in RPA-ProMem - From T1 to T2: < improvement in RAVLT total learning score and delayed recall and appointment memory, n.s.d. in RPA-ProMem
Schwarz et al. (2022)	*N *= 7 PWE; 47.29 ± 8.43 years; 57% women	Drug-resistant left TLE and hippocampal sclerosis	Surgery: resection of the anterior temporal lobe and the anterior hippocampus ASMs	Cognitive behavior training: structured intervention directed toward solving current problems and teaching skills to modify dysfunctional thinking and behavior. Therapy focus was set in accordance with the cognitive deficits and client limitations Strategies: internal and external compensatory Use of brain training games: no Intervention format: individual	Sessions of 45 min two times a week, with at least four sessions before surgery and for at least six months after surgery (total: seven months); total: 42 h	Not included	Not included	3–6 months before surgery (T0), and one week (T1), three months (T2), and six months after surgery (T3) Intervention started 1 month before surgery	- BNT - Regensburger Wortflüssigkeits test - Berliner Amnesie test - Digit span test	*Change after the intervention from baseline:* - From T0 to T1 (after surgery): ↓ naming - From T0 to T2: ↓ verbal memory and verbal fluency - From T2 to T3: ↑ verbal memory and verbal fluency
Sharma et al. (2024)	EG: *n *= 15 PWE; 28.06 ± 8.54 years; 33% women CG: *n *= 13 PWE; 26.61 ± 8.36 years; 31% women	Drug-resistant epilepsy	Some patients underwent surgery, and the others were not suitable candidates ASMs	EG: TAU + EMPOWER: psychoeducation, compensatory training, and cognitive retraining: 126 progressively difficult paper–pencil retraining tasks for attention, verbal memory, and visual memory (42 tasks for each domain) Strategies: internal and external compensatory Use of brain training games: no Intervention format: individual	Eight sessions: two sessions at the hospital (up to 3 h) for psychoeducation and compensatory strategies, five sessions at home with video call and caregiver assistance (30–45 min each) with homework, one session of 30–45 min at home with video call for assessment of compliance and revision of environmental modification and use of internal and external aids; total duration: 6–7.5 h (mean: 6.75 h)	CG: TAU	-	Baseline (T0) and 6–7 months after the intervention (T1)	- AVLT	*EG vs CG:* - From T0 to T1: > ↑ learning, immediate recall, delayed recall, long-term retention
Streltzov et al. (2022)	EG1: *n *= 20 PWE; 48.40 ± 12.50 years; 75% women EG2: *n *= 17 PWE; 44.20 ± 9.90 years; 71% women CG: *n *= 48 PWE; 48.20 ± 11.60 years; 65% women	Controlled epilepsy: - EG1: *n *= 14 - EG2: *n *= 12 - CG: *n *= 32 Idiopathic generalized epilepsy: - EG1: *n *= 6 - EG2: *n *= 5 - CG: *n *= 8	ASMs: stable regimen for 1 month prior to enrolment Prior surgery: - EG1: *n *= 7 - EG2: *n *= 5 - CG: *n *= 13	EG1 and EG2: HOBSCOTCH by a trained coach: patient education, self-monitoring training, problem-solving therapy, and memory strategies EG1: session 1 and 8: in person; rest of the sessions: by telephone EG2: session 1 and 8: virtual; rest of the sessions: by telephone Strategies: internal and external compensatory Use of brain training games: no Intervention format: individual	Eight sessions of 45 to 60 min over 8 to 12 weeks; total: 6–8 h (mean: 7 h)	CG: no intervention (waiting list)	-	EG1 and EG2: Baseline (T0), at 3 months (T1), 6 months (T2), 9 months (T3) and 12 months (T4) after the intervention CG: Baseline (T0), at 3 months (T1), and 6 months (T2) after the intervention in the EGs	- BTACT: word list encoding and recall, digit forward, sequencing, and category fluency	*EG1 and EG2 vs CG:* - n.s.d. in BTACT at any time points
Thompson et al. (2016)	EG1: *n *= 22 PWE; 44.70 ± 8.1 years; 36% women EG2: *n *= 19 PWE; 43.80 ± 9.70 years; 42% women EG3: *n *= 17 PWE; 42.50 ± 11.50 years; 35% women CG: *n *= 19 PWE; 42.40 ± 13.30 years; 42% women	TLE	ASMs (15 different types) in all patients except for 3 Surgery: -EG1: *n *= 8 -EG2: *n *= 8 -EG3: *n *= 8 -CG: *n *= 9	EG1: traditional memory training: use of external aids (calendars, alarms, apps, diaries), internal strategies (visual imagery, Method of Loci, Story Method, and first-letter mnemonics) EG2: Lumosity: memory, attention, speed, and problem-solving games EG3: traditional memory training + Lumosity Strategies: internal and external compensatory (EG1 and EG3) + restorative (EG2 and EG3) Use of brain training games: yes (EG2 and EG3) Intervention format: individual	EG1 and EG3: memory training: 2 sessions; total: 4 h EG2 and EG3: Lumosity: total estimated duration: 45 h Mean duration (EG1, EG2 and EG3): 32.67 h	CG: no intervention	-	Baseline (T0) and immediately post-intervention (T1)	- BMIPB: list Learning and Story Recall tests	*EG1 vs CG:* -From T0 to T1: > ↑ verbal recall; RCIs: n.s.d. in frequencies of improvement, no changes or declines *EG2 vs CG:* -From T0 to T1: n.s.d. in cognitive change *EG3 vs CG:* -From T0 to T1: > ↑ verbal recall; RCIs: more participants with clinical improvements in verbal learning *EG2 and EG3:* -n.s.r. between the number of days or games played and memory performance

*Instrument citations.* AVLT (Rao et al., 2004); Berliner Amnesie test (Metzler et al., 2010); BMIPB (Coughlan et al., 2007); BNT (Kaplan et al., 1983); BTACT (Tun & Lachman, 2006); BVMT-R (Benedict et al., 1996); CDT (Ferrucci et al., 1996; Shulman et al., 1993); COWA (Strauss et al., 2006); CPT (Conners, 1995); DCS (Lamberti & Weidlich, 1999); Digit span forward and backward (Wechsler, 1997); Digit Vigilance Test (Lezak, 1995); DSST (Lezak et al., 2004; Wechsler, 1981); GDT (Brand et al., 2006); IGT (Bechara et al., 1997); Letter cancelation test (Brickenkamp, 1978); MMSE (Folstein et al., 1975); N back test (Smith & Jonides, 1999); Phonemic and semantic fluency (Novelli et al., 1986); Raven Progressive Matrices (Basso et al., 1987; Caffarra et al., 2003); RAVLT (Carlesimo et al., 1996; Rey, 1941, 1958; Schmidt, 1996); RBANS (Randolph et al., 1998); Recall of disyllabic words (Spinnler & Tognoni, 1987); Regensburger Wortflüssigkeits test (Aschenbrenner et al., 2000); Rey-Osterreith Word List (Carlesimo et al., 1996); ROCF (Caffarra et al., 2002; Carlesimo et al., 2002; Osterrieth, 1944); Span forward and backward (Orsini et al., 1987); SPART (Boringa et al., 2001); SRT (Ehrenreich, 1995); Story recall (Carlesimo et al., 2002); Stroop test (Golden, 1978; Strauss et al., 2006); TMT A and B (Giovagnoli et al., 1997; Strauss et al., 2006); Tower of London (Krikorian, 1994; Shallice, 1982); Verbal Fluency Test (Lezak et al., 2004); Visual search (Spinnler et al., 1987); VLMT (Helmstaedter et al., 2001); WAIS-IV (Egeland, 2015); WCST (Heaton, 1981; Milner, 1963). Abbreviations. *ASMs* antiseizure medications; *AVLT* Auditory Verbal Learning Test; *BMIPB* BIRT Memory Information Processing Battery; *BNT* Boston Naming Test; *BTACT* Brief Test of Adult Cognition by Telephone; *BVMT-R* Brief Visuospatial Memory Test-Revised; *CDT* clock-drawing test; *CG* control group; *CogSMART* Cognitive Symptom Management and Rehabilitation Training; *COWA* Controlled Oral Word Association; *CPT* Corner’s Continuous Performance Test; *DCS* Diagnostikum für Zerebralschädigung; *DSST* Digit Symbol Substitution Test; *EG* experimental group; *EMPOWER* Indigenized Home-Based Attention and Memory Rehabilitation Program for Adult Patients with Drug Refractory Epilepsy); *FLE* frontal lobe epilepsy; *fMRI* functional magnetic resonance imaging; *GDT* Game of Dice Task; HOBSCOTCH HOme Based Self-management and COgnitive Training CHanges lives; *IGT* Iowa Gambling Task; MMSE Mini-Mental State Examination; *n.s.* not specified; *n.s.d.* no significant differences; *n.s.r.* no significant relationship; *OLE* occipital lobe epilepsy; *PWE* patients with epilepsy; *RAVLT* Rey Auditory Verbal Learning Test; *RBANS* Repeatable Battery for the Assessment of Neuropsychological Status; *RCI* Reliable Change Index; *ROCF* Rey-Osterrieth Complex Figure; *RPA-ProMem* Royal Prince Alfred Prospective Memory Test; *SPART* 10/36 Spatial Recall Test; *SRT* Selective Reminding Test; *TAU* treatment as usual; *TLE* temporal lobe epilepsy; *TMT* Trail Making Test; *VLMT* Verbaler Lern und Merkfähigkeitstest; *VR* virtual reality; *WAIS-IV* Wechsler Adult Intelligence Scale-4th Edition; *WCST* Wisconsin Card Sorting Test

**Table 2:** Summary of studies with pediatric patients

Reference	Sample size; mean age ± SD; % of women	Epilepsy type	Epilepsy treatment	Experimental intervention		Control intervention		Assessment time	Neuropsychological assessment instruments	Results
				Characteristics of intervention	Number of sessions and duration	Characteristics of intervention	Number of sessions and duration			
Fuentes & Kerr (2017)	*N *= 28 PWE; 10.81 ± 2.51 years; 57.14% girls	n.s	ASMs	CogMed “RoboMemo” program: 12 working memory exercises (7 visual-spatial, 2 visual-verbal, and 3 auditory with visual responses), each session consisting of 8 exercises Level of difficulty adjusted to the child’s performance Strategies: restorative Use of brain training games: yes Intervention format: individual	Five sessions per week during a 5- to 7-week period; 30–45 min each session, total: 2.5–3.75 h (mean 3.13 h)	Not included	Not included	T0: one week before the intervention T1: within one week post-intervention (i.e., approximately 7 weeks after T0) T2: 3-month follow-up visit (T2)	- Backward Digit Recall and Counting Recall from the WMTB-C - Spatial Span Forward and Spatial Span Backward from the WISC-IV - The ACT - The Matrix Reasoning subtest from the WASI-II	*From T0 to T1:* - ↑ in verbal and visual attention and working memory - n.s.d. in visual-verbal working memory or fluid reasoning - RCIs: 36% to 64.29% of participants had improvements *From T0 to T2:* - Gains remained stable, with the latter scores being higher than at T0 -RCIs: 24% to 53.57% of participants had improvements
Glynn et al. (2016)	*N *= 8 PWE; 9.8 ± 1.7 years; 50% girls	Childhood absence epilepsy	ASMs	Constant Therapy (software for iPad). Tasks for training attention or working memory Each participant began the same tasks at the same level of difficulty. After six tasks, task difficulty and type were adjusted according to individual performance Strategies: restorative Use of brain training games: yes Intervention format: individual	Four sessions per week, 20 min per day, for 4 weeks; total: 5.33 h	Not included	Not included	Baseline and within a week of completion of the intervention	Cognitive domain of the NIH Toolbox: picture vocabulary, flanker inhibitory control and attention, list sorting working memory, dimensional change card sort, pattern comparison process speed, picture sequence memory, and oral reading recognition	*After intervention:* - ↑ in processing speed and fluid intelligence
Kerr and Blackwell (2015)	EG: *n *= 42 PWE; 11.1 ± 3.0 years; 42.9% girls CG: *n *= 35 PWE; 10.6 ± 2.7 years; 54.3% girls From those patients, the following were not included: EG: *n *= 5 PWE; 11.7 ± 3.2 years; 20% girls CG: *n *= 14 PWE; 10.9 ± 2.8 years; 57.1% girls	Symptomatic epilepsy	ASMs	CogMed “RoboMemo” program: 12 working memory exercises (seven visual-spatial, two visual-verbal, and three auditory with visual responses), with each session consisting of eight exercises Level of difficulty adjusted to the child’s performance Strategies: restorative Use of brain training games: yes Intervention format: individual	Five sessions per week during 5- to 7-week period; 30–45 min each session, total: 2.5–3.75 h (mean: 3.13 h)	CG: no intervention (waiting list)	-	Baseline (T0) and after intervention (approximately 7 weeks apart) (T1)	- Digit Recall Forward, Digit Recall Backward, Listening Recall, and Counting Recall subtests from the WMTB-C - Spatial Span Forward and Backward; and Visual Digit Span subtests from the WISC-IV - Creature Counting subtest and the Score! Subtest of the TEA-Ch - ACT - CPT-II	*From T0 to T1:* - EG vs CG: RCIs: > improvements in listening recall, digit recall backward, spatial span backward, counting recall - EG: ↑ digit recall backward, counting recall, and spatial span forward (even replicating the analyses without the patients who did not undergo follow-up)
Khaleghi et al. (2024)	EG1: *n *= 15 PWE; 9.84 ± 2.37 years; gender: n.s EG2: *n *= 15 PWE; 9.12 ± 2.90 years; gender: n.s CG: *n *= 15 PWE; 10.32 ± 2.63 years; gender: n.s	Controlled epilepsy with ASMs	ASMs	Interventions supervised by a researcher EG1: cognitive rehabilitation program of the CANTAB: spatial working memory and attention shift EG2: beta task-oriented cognitive rehabilitation program: aimed to train sustained attention using paper and pencil. Different levels of difficulty Strategies: internal compensatory + restorative Use of brain training games: yes (EG1) Intervention format: individual	- Computerized cognitive rehabilitation program of the CANTAB: 12 sessions of 45 min; total: 9 h - Task-oriented cognitive rehabilitation program: 12 sessions of 45 min for three months; total: 9 h	CG: no intervention	-	Baseline (T0), after the intervention (T1), and follow-up (T2)	- IVA2/CPT	*From T0 to T1 and T2:* *-* ↑ in attention in EG1 and EG2 - > scores in attention in the EG1 and EG2 than in the CG -n.s.d in attention performance between EG1 and EG2
Saard et al. (2017)	EG: *n *= 17 PWE and attention impairment; 9.95 years, SD: n.s., range: 9.33–10.57; 29% girls CG1: *n *= 12 PWE and attention impairment; 10.20 years; SD: n.s.; range: 9.08–11.32; 25% girls CG2: *n *= 19 typically developing children; mean age ± SD: n.s., range: 8–12 years; 42% girls	EG and CG1: partial epilepsy	EG and CG1: ASMs	EG: FORAMENRehab computer-based intervention accompanied by a therapist: attention module: focused attention, sustained attention, complex attention, and tracking. Use of interactive, short exercises (1–4 min, up to 20 min for sustained attention) with an intuitive interface, clear instructions, and customizable task settings. Therapist guidance and metacognitive support Strategies: restorative + internal compensatory Use of brain training games: yes Intervention format: individual	10 sessions of 40 min over 5 weeks (twice a week); total: 6.67 h	CG1: no intervention (waiting list) CG2: no intervention	-	EG and CG1: Baseline (T0), immediately after the intervention (T1), and follow-up at 1.31 years (T2) CG2: Only baseline (T0)	- Correct responses, omission and commission errors, total number of mistakes, reaction time, and processing speed for each task (focused attention, sustained attention, complex attention, and tracking) of the FORAMENRehab software	*EG vs CG1:* -T1: > performance in Paced Search with Dual Targets (complex attention), Word Recognition with Dual Targets (complex attention) Addition task (complex attention) and PASAT (tracking component) -T1: n.s.d. in focused and sustained attention -T2: > performance in Paced Search with Dual Targets (complex attention), Addition task (complex attention) and PASAT (tracking component) *EG and CG1 vs CG2:* -T0: < performance in sustained attention, complex attention, and tracking *EG:* -From T0 to T1: ↑ complex attention and tracking components -From T0 to T2: ↑ focused attention, all complex attention tasks, and tracking *CG1:* -From T0 to T1: no significant changes -From T0 to T2: ↑ in two measures of complex attention
Saard et al. (2019)	EG: *n *= 17 PWE and attention impairment; 9.95 ± 1.21 years; 29% girls CG1: *n *= 22 PWE and attention impairment; 10.29 ± 1.85 years; 32% girls CG2: *n *= 19 typically developing children; mean age ± SD: n.s., range: 8–12 years; 42% girls	EG and CG1: partial epilepsy	EG and CG1: ASMs	EG: FORAMENRehab computer-based intervention accompanied by a therapist: visuospatial module: visual recognition, visual organization, visual attention, and visuospatial perception. Use of interactive, short exercises (up to 5 min) with an intuitive interface, clear instructions, and customizable task settings Therapist guidance and metacognitive support Strategies: restorative + internal compensatory Use of brain training games: yes Intervention format: individual	10 sessions of 40 min over 5 weeks (twice a week); total: 6.67 h	CG1: no intervention (waiting list) CG2: no intervention	-	EG and CG1: Baseline (T0), immediately after the intervention (T1), and follow-up at 1.31 years (T2) CG2: Only baseline (T0)	- Solving and/or reaction time, number of correct responses, and mistakes for each task: Circle Following and Line Orientation Judgement (visual recognition), Geometric Pattern Recognition (visual organization), Spatial Attention (visual attention), and Cubes, Construction and Maze (visuospatial perception) of the FORAMENRehab software	*EG and CG1 vs CG2:* -T0: < performance in Line Orientation Judgment, Geometric Pattern Recognition, Spatial Attention, Cubes, Construction, and Maze *EG:* -From T0 to T1: ↑ Geometric Pattern Recognition, Spatial Attention, Cubes, and Construction -From T0 to T2: ↑ Line Orientation Judgment, Geometric Pattern Recognition, Spatial Attention, Construction, and Maze *CG1:* -From T0 to T1: n.s -From T0 to T2: ↑ Geometric Pattern Recognition
Schaffer et al. (2017)	EG: *n *= 33 PWE; 10.88 ± 1.50 years; 51% girls CG: *n *= 27 healthy children; 10.18 ± 1.40 years; 52% girls	EG: drug-resistant epilepsy, including self-limited rolandic epilepsy (*n *= 18) and genetic generalized epilepsy (*n *= 15)	ASMs	EG: Two modules: Memory Skills Training: chunking and mnemonic methods, self- awareness of individual memory slips, categorization, story making, visual imagery, and association + smartphone reminders, routines and habits; and psychosocial training. “homework” task completion Strategies: internal and external compensatory Use of brain training games: no Intervention format: group-based	10 sessions over 10 weeks; total: 15 h	CG: no intervention	-	Baseline and immediately post-intervention (within 1–2 weeks from the end of the last group session)	- TOMAL: paired recall, object recall, immediate and delayed word selective memory, immediate and delayed memory for stories, digit forward and backward, facial memory, abstract visual memory, visual sequential memory, memory for location -CMS: immediate and delayed memory -RAVLT: immediate and delayed memory -ROCF: immediate and delayed visual memory -NEPSY: inhibition, animal sorting - WISC-V: cancellation	*EG vs CG:* -Higher proportion of participants having short-term auditory verbal memory at normal range at baseline and post-intervention *EG:* - ↑ short-term memory −21% of the children shift from deficient levels to normal range in auditory verbal memory -Children with lower executive function scores gained more from the intervention than those with higher executive function scores in verbal tasks
Tapia et al. (2024)	*N *= 26 PWE; 12.12 ± 2.96 years; 46% girls	- Drug-resistant focal epilepsy: *n *= 2 - Drug-resistant epilepsy: *n *= 6 - Focal epilepsy: *n *= 2 - Dravet Syndrome: *n *= 3 - Genetic epilepsy: *n *= 1 - Idiopathic epilepsy: *n *= 1 - Rolandic epilepsy: *n *= 2 -Generalized epilepsy: *n *= 1 -Unspecified epilepsy: *n *= 8	ASMs	CogniFit Inc. platform: personalized tasks aiming to improve inhibitory skills Strategies: restorative Use of brain training games: yes Intervention format: individual	40 sessions of 12–15 min over 16 weeks (with a minimum of 3 sessions per week); total: 8–10 h (mean: 9 h)	Not included	Not included	Baseline and immediately after the intervention	- Adapted version of the anti-saccade test from the DIGICOG executive function battery (Impulso Cognitivo SL, Ermua, Spain)	*Change after the intervention from baseline:* - ↓ reaction times in the anti-saccade test (better inhibitory control)

*Instrument citations.* ACT (Weber & Segalowitz, 1990); CMS (Cohen, 1997); CPT-II (Conners, 2009); IVA2/CPT (Sandford & Turner, 2000); NIH Toolbox (Gershon et al., 2010); NEPSY (Korkman et al., 2007); RAVLT (Carlesimo et al., 1996; Rey, 1941, 1958; Schmidt, 1996); ROCF (Caffarra et al., 2002; Carlesimo et al., 2002; Osterrieth, 1944); TEA-Ch (Manly et al., 1999); WASI-II (Wechsler, 2012); WISC-IV (Wechsler et al., 2004); WISC-V (Wechsler, 2003); WMTB-C (Pickering & Gathercole, 2001). Abbreviations. *ACT* Attentional Capacity Test; *ASMs* antiseizure medications; *CANTAB* Cambridge Neuropsychological Test Automated Battery; *CG* control group; *CMS* Child memory scale; *CPT* Corner’s Continuous Performance Test; *EG* experimental group; *IVA2/CPT* Integrated Visual and Auditory Continuous Performance Test; *n.s.* not specified; *n.s.d.* no significant differences; *PWE* patients with epilepsy; *RAVLT* Rey Auditory Verbal Learning Test; *RCI* Reliable Change Index; *ROCF* Rey-Osterrieth Complex Figure; *TEA-Ch* Test of Everyday Attention for Children; *TOMAL* test of memory and learning (Reynolds & Bigler 1994); *WASI-II* Wechsler abbreviated scale of intelligence-2nd Edition; *WISC* Wechsler Intelligence Scale for Children; *WMTB-C* Working Memory Test Battery for Children

During the selection process, the interrater agreement between reviewers was 86.1%. Specifically, 11 instances of disagreement between reviewers were identified in the revision of titles and abstracts. Of these 11 cases, 10 studies were retained for full-text review, and one was excluded. Following full-text assessment, two of the 10 studies were ultimately included. For the data extraction, the level of interrater agreement between reviewers was 96.5%. Specifically, disagreements were identified in the following categories: sample size (1 article; 0.5%), characteristics and number of sessions of the experimental intervention (5 articles; 2.5%), and assessment time (1 article; 0.5%). It is noteworthy that a single article (Grewe et al., 2013) presented discrepancies in three separate items. Discrepancies were resolved through discussion until 100% of agreement was reached.

### Evaluation of the Quality of the Studies

Following the JBI criteria to assess the quality of the studies included in this systematic review (Table 3), it was observed that all the reviewed studies clearly distinguished between cause and effect, ensuring a lack of ambiguity about which variable preceded the other. Sixteen out of 25 studies (64.0%) included a control group. Twelve studies (48.0%) considered participant groups with similar characteristics. In twenty-four of the studies (96.0%), participants received comparable treatment, regardless of the specific intervention. Twenty-two studies (88.0%) included multiple measurements of the outcome (before and after the intervention), used reliable methods to measure the outcomes, and used appropriate statistical analyses. Twenty-four studies (96.0%) used the same measures to assess the outcomes across comparisons and completed a follow-up or described and properly analyzed group differences for follow-ups. According to the levels of evidence classification system (Melnyk & Fineout-Overholt, 2022), five studies fall under level 2, seven under level 3, nine under level 4, and four under level 6 (Table 3).

**Table 3:** Methodological quality assessment and classification according to the levels of evidence

	Critical appraisal checklist									Level of evidence
Authors	Q1	Q2	Q3	Q4	Q5	Q6	Q7	Q8	Q9	
Anuradha et al. (2014)	Y	Y	Y	Y	Y	Y	Y	Y	Y	3
Caller et al. (2016a)	Y	Y	Y	Y	Y	Y	Y	Y	Y	2
de Vasconcelos et al. (2017)	Y	Y	Y	Y	Y	Y	Y	Y	Y	3
Fuentes & Kerr (2017)	Y	N	U	Y	Y	Y	Y	Y	Y	4
Gaston et al. (2019)	Y	N	U	Y	Y	Y	Y	Y	Y	4
Glynn et al. (2016)	Y	N	U	Y	Y	Y	Y	Y	Y	4
Grewe et al. (2013)	Y	Y	U	N	N	N	U	Y	Y	6
Helmstaedter et al. (2008)	Y	Y	Y	Y	Y	Y	Y	Y	Y	3
Icellioglu et al. (2017)	Y	N	U	Y	Y	Y	Y	Y	N	6
Jones (1974)	Y	Y	U	Y	N	Y	U	Y	Y	6
Kerr and Blackwell (2015)	Y	Y	U	Y	Y	Y	Y	Y	Y	2
Khaleghi et al. (2024)	Y	Y	U	Y	Y	Y	Y	Y	N	4
Koorenhof et al. (2012)	Y	Y	Y	Y	Y	Y	Y	Y	Y	3
Maialetti et al. (2020)	Y	Y	N	Y	Y	Y	Y	Y	Y	3
Maschio et al. (2015)	Y	N	U	Y	Y	Y	Y	Y	Y	4
Paiva et al. (2023)	Y	N	U	Y	Y	Y	Y	Y	Y	4
Radford et al. (2011)	Y	N	Y	Y	Y	Y	Y	Y	Y	4
Saard et al. (2017)	Y	Y	Y	Y	Y	Y	Y	Y	Y	3
Saard et al. (2019)	Y	Y	Y	Y	Y	Y	Y	Y	Y	3
Schaffer et al. (2017)	Y	Y	Y	Y	U	Y	Y	Y	Y	4
Schwarz et al. (2022)	Y	N	U	Y	Y	Y	Y	Y	N	6
Sharma et al. (2024)	Y	Y	Y	Y	Y	Y	Y	U	Y	2
Streltzov et al. (2022)	Y	Y	Y	Y	Y	Y	Y	Y	Y	2
Tapia et al. (2024)	Y	N	U	Y	Y	Y	U	Y	Y	4
Thompson et al. (2016)	Y	Y	Y	Y	Y	Y	Y	Y	Y	2

N: no; U: unclear; Y: yes; Q1: Is it clear in the study what is the “cause” and what is the “effect”? (i.e. there is no confusion about which variable comes first); Q2: Was there a control group?; Q3: Were participants included in any similar comparisons?; Q4: Were the participants included in any comparisons receiving similar treatment/care, other than the intervention of interest?; Q5: Were there multiple measurements of the outcome, both pre and post the intervention/exposure?; Q6: Were the outcomes of participants included in any comparisons measured in the same way?; Q7: Were outcomes measured in a reliable way?; Q8: Was follow-up complete and if not, were differences between groups in terms of their follow-up adequately described and analyzed?; Q9: Was appropriate statistical analysis used? Methodological quality assessment is based on the Critical appraisal checklist for quasi-experimental studies (Barker et al., 2024), and levels of evidence follow the classification system of Melnyk and Fineout-Overholt (2022)

The interrater agreement between reviewers was 97.8%, with discrepancies in five out of 225 responses (2.2%), which were addressed through discussion and resolved by consensus.

### Qualitative Analysis of the Reviewed Studies

#### Characteristics of the Sample

Regarding the age group, 17 of the 25 studies (68.0%) included a sample of adults with epilepsy (Table 1), whereas the remaining eight studies (32.0%) included children or adolescents with epilepsy (Table 2).

With respect to epilepsy type, among the 17 studies conducted with adult samples, 13 specified whether participants had focal or generalized epilepsy. Of these 13 studies, seven (53.8%) included samples with focal epilepsies, with five of these seven studies (71.4%) including samples with temporal lobe epilepsy (TLE). Two studies (15.4%) included samples with generalized epilepsies, whereas four studies (30.8%) included mixed samples of patients with focal and generalized epilepsies. Four studies did not specify whether the sample had focal or generalized epilepsy. Regarding seizure control, six of the 17 adult studies (35.3%) included samples composed exclusively of patients with drug-resistant epilepsy, and the remaining studies did not provide information. Among studies with pediatric samples, five studies specified whether the sample had focal or generalized epilepsy: two of these five studies (40.0%) included samples with focal epilepsies, one study (20.0%) included generalized epilepsies, whereas two studies (40.0%) included mixed samples of focal and generalized epilepsies. Three studies did not specify whether the sample had focal or generalized epilepsy. Regarding seizure control, one pediatric study included only patients with drug-resistant epilepsy, another included only patients with controlled epilepsy, and the rest did not report this information.

Concerning epilepsy treatment, among the 17 studies involving adult patients, six (35.3%) reported that the sample was treated with antiseizure medications (ASMs); in six studies (35.3%), all patients were treated with ASMs and surgery, and in five studies (29.4%), patients were treated with ASMs and a variable percentage underwent surgery. All studies involving pediatric patients reported that participants were treated exclusively with ASMs.

#### Interventions Characteristics

Experimental interventions can be classified depending on the use of restorative strategies based on brain training games, the use of compensatory strategies, and the intervention format (i.e., individual or group-based) (Tables 1 and 2).

Seven studies of the 17 studies with adult samples (41.2%) used brain training games such as Brain Age and Lumosity, whereas 10 studies (58.8%) did not employ brain training games. Among studies with pediatric samples, seven out of eight (87.5%) employed brain training games such as CogMed “RoboMemo,” CogniFit, and FORAMENRehab, while only one study did not.

Regarding the use of compensatory strategies, two of the 17 adult studies (11.8%) employed exclusively internal compensatory strategies, whereas 12 studies (70.6%) used both internal and external compensatory strategies, and three (17.6%) did not use compensatory strategies. In studies with pediatric samples, three of the eight studies (37.5%) employed exclusively internal compensatory strategies, one study (12.5%) combined internal and external compensatory strategies, and the remaining four (50.0%) did not use compensatory strategies.

Regarding the intervention format, 15 out of 17 studies with adult samples (88.2%) primarily used an individual format—12 exclusively individual and three predominantly individual with some group-based sessions—while the remaining two studies (11.8%) employed a group-based format. Among the pediatric studies, seven out of eight (87.5%) used an individual intervention format.

The average duration of the experimental interventions in studies with adult samples was 15.86 h (SD = 12.94), with values ranging from 2 to 44 h. Specifically, eight studies included interventions lasting up to 10 h, five studies lasting more than 10 and up to 20 h, and four studies exceeding 20 h. In studies with pediatric patients, the average duration of the experimental interventions was 7.24 h (SD = 3.87), and duration ranged from 3.13 to 15 h. Specifically, seven studies included interventions lasting up to 10 h, and only one lasting more than 10 and up to 20 h.

Among the 17 studies with adult patients, 11 (64.70%) included a control group. Among these 11 studies, eight included a control group of patients with epilepsy without neuropsychological intervention (e.g., waiting list). Three of these 11 studies included a control group of healthy participants: two with the same neuropsychological intervention as patients with epilepsy, one without intervention, and one with a control intervention that consisted of memory training without computerized brain training. Six of the 17 adult studies (35.30%) did not include a control group. Among the eight studies with pediatric samples, five (62.50%) included control groups: four of them had a control group of patients with epilepsy without neuropsychological intervention (e.g., waiting list), and three had a control group of healthy participants without intervention—note that some studies included both types of control groups. Three of the eight pediatric studies (37.50%) did not include a control group.

#### Neuropsychological Assessment

Data about neuropsychological assessment is shown in Tables 1 and 2. Eleven of the 17 studies with adult samples (64.7%) performed a neuropsychological assessment before the intervention and only one post-intervention assessment. Four of the 17 studies (23.5%), in addition to pre- and post-intervention assessments, included one or more follow-ups. The remaining two studies (11.8%) conducted assessments during the intervention (using performance on the intervention tasks as the assessment instrument). Among the eight studies with pediatric samples, four (50.0%) performed a neuropsychological assessment before the intervention and only one post-intervention assessment, and the remaining four (50.0%) also included one or more follow-ups.

Regarding attention, seven of the 17 adult studies (41.2%) assessed intervention effects on this outcome, with the most frequently used tests being the Trail Making Test A and both letter and color trials of the Stroop Test. Among studies with pediatric samples, seven of the eight studies (87.5%) examined intervention effects on attention, with the most frequently used tests being the Attentional Capacity Test and a Continuous Performance Test.

For executive functions, five of the 17 adult studies (29.4%) examined intervention effects on this outcome, with the most frequently used tests being the Trail Making Test B, the Wisconsin Card Sorting Test, the Tower of London Test, and the interference trial of the Stroop. Four of the eight pediatric studies (50.0%) evaluated intervention effects on executive function, with the most frequent tests being sorting tasks.

Working memory was evaluated in seven of the 17 adult studies (41.2%), with the most frequently used test being a digit span task and the Digit Symbol Substitution Test. Among the eight pediatric studies, five (62.5%) evaluated working memory, primarily using digit span tasks, as well as spatial span and counting recall tasks.

Regarding immediate episodic memory, 15 of the 17 studies with adult samples (88.2%) examined intervention effects on this outcome. These studies also assessed delayed episodic memory, except for studies by de Vasconcelos et al. (2017). The most frequently used tests were the Rey Auditory Verbal Learning Test, the Rey-Osterrieth Complex Figure, and the BIRT Memory Information Processing Battery. Among the eight studies with pediatric samples, only two evaluated immediate episodic memory and one evaluated delayed episodic memory, using the Rey Auditory Verbal Learning Test, the Rey-Osterrieth Complex Figure, and a picture sequence memory task.

Language was evaluated in ten of the 17 adult studies (58.8%). The most frequently used tests were semantic fluency, phonemic fluency, and naming tests. Only one of the eight studies with pediatric samples (12.50%) evaluated language using a naming test.

### Meta-analysis of Pre-post Changes in Experimental Interventions

#### Studies with Adult Patients

We included 13 studies (251 participants) in the multivariate meta-analysis examining pre-post changes in experimental interventions in adult patients. Icellioglu et al. (2017) provided the scores of the word and color sections of the Stroop test separately, so a global score was calculated based on both sections. Maschio et al. (2015) and Paiva et al. (2023) provided both forward and backward digit span scores separately, so an overall score was calculated for both tasks as an indicator of working memory.

**Fig. 2:**
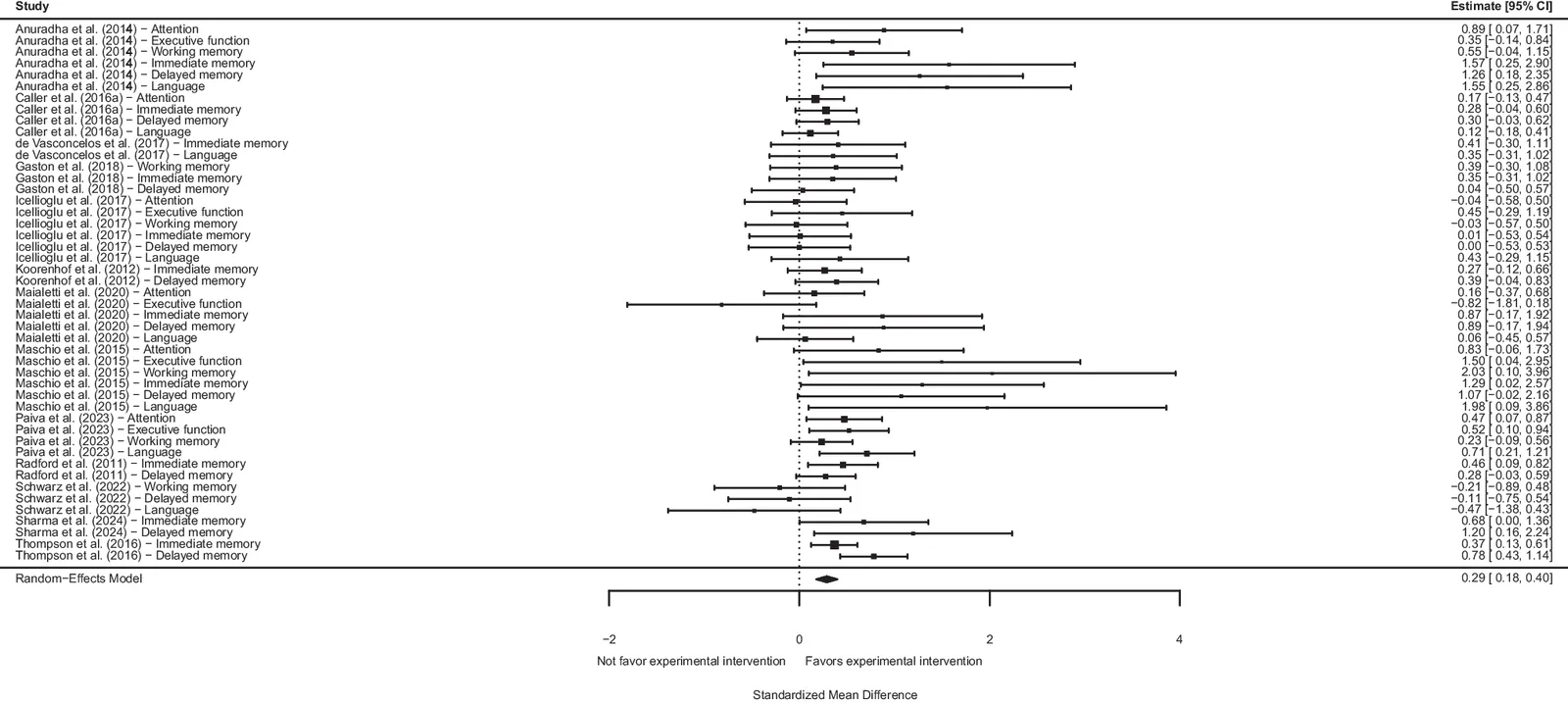
Forest plot for the effectiveness of neuropsychological interventions in improving cognitive function in adult patients (overall effect)

The summary effect demonstrated a significant overall positive effect of experimental interventions on cognition (*g* = 0.29; 95% CI = 0.18, 0.40; *p* < 0.0001) (Fig. 2 and Table 4). Heterogeneity was not significant (*Q*(46) = 60.50, *p* = 0.07; attention: *τ*^2^ = 0.00; *I*^2^ = 4.2%; executive functions: *τ*^2^ = 0.17; *I*^2^ = 45.1%; working memory: *τ*^2^ = 0.00; *I*^2^ = 1.7%; immediate memory: *τ*^2^ = 0.00; *I*^2^ = 1.6%; delayed memory: *τ*^2^ = 0.04; *I*^2^ = 21.3%; language: *τ*^2^ = 0.03; *I*^2^ = 10.0%). Two studies were identified as potential outliers, with absolute studentized deleted residuals greater than 1.96: Thompson et al. (2016) (*z* = 2.46) and Maialetti et al. (2020) (*z* = 2.06). Cook’s distance values were highest for Icellioglu et al. (2017) (*D* = 0.13) and Caller et al. (2016a) (*D* = 0.11), indicating a moderate influence on the overall model. However, DFBETAs did not exceed the threshold of ± 1, suggesting no single study had a disproportionate impact on the overall effect size estimate. No study was identified as an influential case across all diagnostics, so all studies were retained in the subsequent analyses, and the influence of potential moderators was examined. Egger’s regression intercept suggested potential small-study effects (Intercept = 0.65; SE = 0.16; 95% CI = 0.33, 0.97; *z* = 4.01; *p* < 0.0001). The adjusted effect size under the three-parameter selection model (*g* = 0.23; 95% CI = 0.00, 0.45; *p* = 0.048) tended to differ from the unadjusted estimate, although differences did not reach statistical significance (LRT = 3.19, *p* = 0.07).

**Table 4:** Effect sizes of multivariate meta-analysis of pre-post changes in experimental interventions in adult patients

Effect size by outcome	*k*	Hedges’ *g*		95% CI		95% prediction intervals	
		*g*	*p*	Lower	Upper	Lower	Upper
Overall effect on cognition	13	0.29	0.0001	0.18	0.40	−0.14	0.71
Cognitive outcome							
Attention	6	0.24	0.0098	0.06	0.43	−0.03	0.51
Executive functions	5	0.04	0.89	−0.46	0.53	−1.24	1.32
Working memory	6	0.17	0.10	−0.03	0.36	−0.06	0.40
Immediate episodic memory	11	0.34	0.0001	0.21	0.47	0.18	0.50
Delayed episodic memory	11	0.38	0.0001	0.18	0.57	−0.09	0.85
Language	8	0.33	0.0159	0.06	0.59	−0.28	0.93

When the cognitive outcome was included as a moderator, the results showed significant variability in effect sizes across cognitive domains (QM(6) = 32.59, *p* < 0.0001). Heterogeneity was not significant (*Q*(41) = 54.54, *p* = 0.08; attention: *τ*^2^ = 0.01; *I*^2^ = 6.2%; executive functions: *τ*^2^ = 0.27; *I*^2^ = 56.4%; working memory: *τ*^2^ = 0.00; *I*^2^ = 0.4%; immediate memory: *τ*^2^ = 0.00; *I*^2^ = 0.5%; delayed memory: *τ*^2^ = 0.04; *I*^2^ = 20.1%; and language: *τ*^2^ = 0.06; *I*^2^ = 18.7%). The effects of experimental interventions were significant for attention (*g* = 0.24; 95% CI = 0.06, 0.43; *p* = 0.0098), immediate episodic memory (*g* = 0.34; 95% CI = 0.21, 0.47; *p* < 0.0001), delayed episodic memory (*g* = 0.38; 95% CI = 0.18, 0.57; *p* < 0.0001), and language (*g* = 0.33; 95% CI = 0.06, 0.59; *p* = 0.0159). However, the effects were not significant for executive functions (*g* = 0.04; 95% CI = −0.46, 0.53; *p* = 0.89) and working memory (*g* = 0.17; 95% CI = −0.03, 0.36; *p* = 0.10). The number of studies for each outcome, the effect sizes, and the prediction intervals are shown in Table 4. Given the significant moderation by cognitive outcome, the subsequent model testing additional moderators also included cognitive outcome to control domain-related variability in effect sizes.

The multivariate meta-regression model examining the influence of study-level moderators revealed no significant overall moderation effect (QM(12) = 13.04, *p* = 0.37), suggesting that the included variables did not explain a significant portion of the residual heterogeneity. Heterogeneity was significant (*Q*(34) = 48.71, *p* = 0.049; attention: *τ*^2^ = 0.01; *I*^2^ = 12.2%; executive functions: *τ*^2^ = 0.34; *I*^2^ = 61.9%; working memory: *τ*^2^ = 0.02; *I*^2^ = 9.4%; immediate memory: *τ*^2^ = 0.00; *I*^2^ = 1.0%; delayed memory: *τ*^2^ = 0.04; *I*^2^ = 22.2%; and language: *τ*^2^ = 0.07; *I*^2^ = 22.3%). No significant differences were found depending on epilepsy treatment (*B* = −0.09; SE = 0.20; 95% CI = −0.49, 0.31; *p* = 0.65), the use of restorative strategies involving brain training games (*B* = −0.34; SE = 0.22; 95% CI = −0.78, 0.10; *p* = 0.13), the use of both internal and external compensatory strategies (*B* = −0.21; SE = 0.39; 95% CI = −0.98, 0.55; *p* = 0.59), the lack of use of compensatory strategies (*B* = 0.21; SE = 0.63; 95% CI = −1.02, 1.44; *p* = 0.74), the individual intervention format (*B* = −0.02; SE = 0.21; 95% CI = −0.42, 0.39; *p* = 0.93), and the intervention duration (*B* = −0.00; SE = 0.08; 95% CI = −0.15, 0.15; *p* = 0.98). However, a higher methodological quality (represented by lower values in the level of evidence classification) was related to better outcomes (*B* = −0.18; SE = 0.09; 95% CI = −0.36, −0.00; *p* = 0.048).

In the above analyses, we assumed a constant correlation of 0.5 between effect sizes within the same study (Cheung, 2019). Sensitivity analyses using alternative correlation values (0 and 0.8) yielded comparable results. The only notable difference was observed when a correlation of 0.8 was applied: the moderating effect of methodological quality, previously significant, did not reach statistical significance (*B* = −0.13; SE = 0.09; 95% CI = −0.32, 0.05; *p* = 0.15). All other outcomes remained consistent across the different correlation assumptions, supporting the robustness of the overall findings.

#### Studies with Pediatric Patients

We included seven studies (163 participants) in the multivariate meta-analysis examining pre-post changes in experimental interventions in pediatric patients. Glynn et al. (2016) used two attentional measures of the NIH toolbox (Gershon et al., 2010), and they were combined in a global score. Kerr and Blackwell (2015) provided both forward and backward digit span scores separately, so an overall score was calculated for both tasks as an indicator of working memory.

**Fig. 3:**
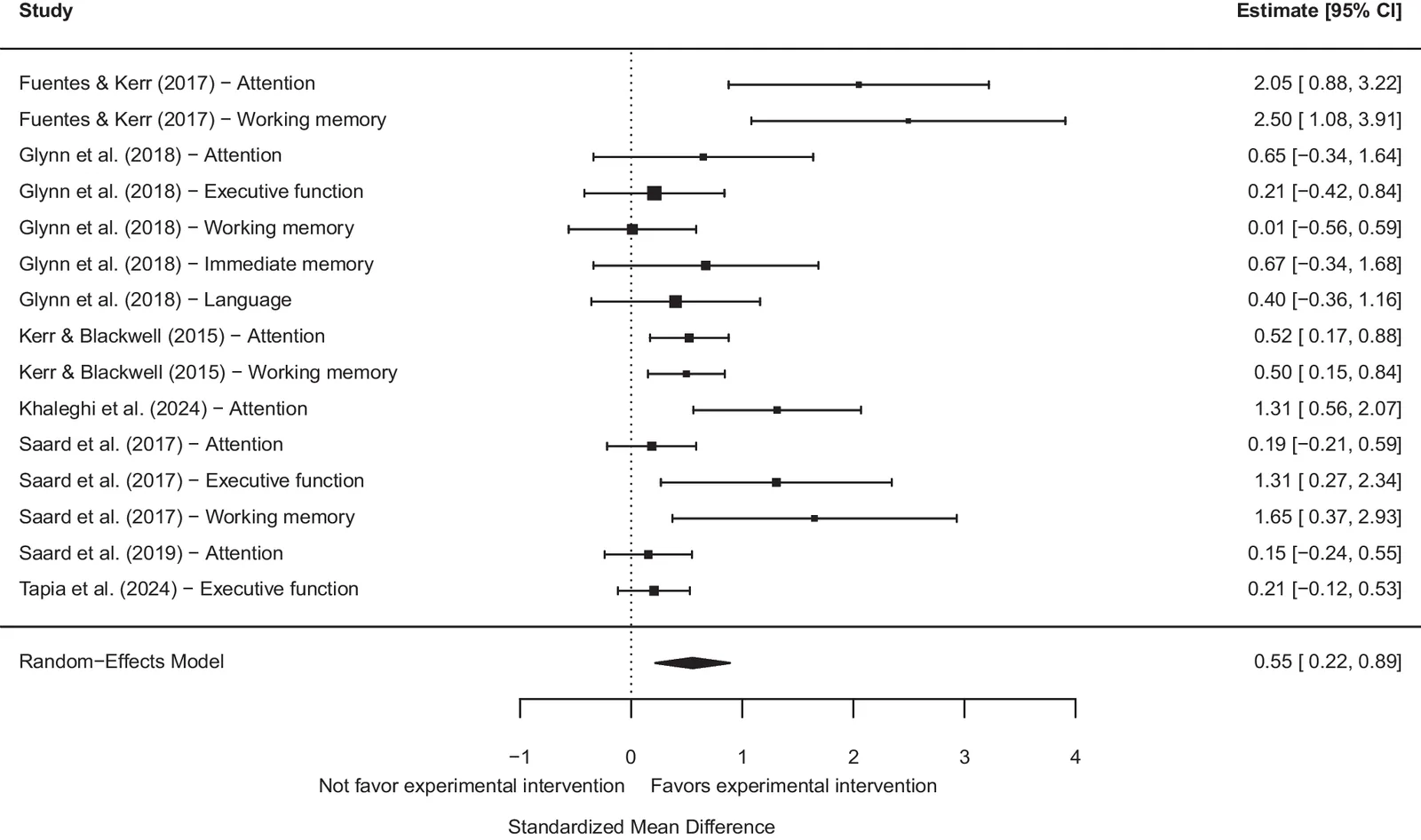
Forest plot for the effectiveness of neuropsychological interventions in improving cognitive function in pediatric patients (overall effect)

The summary effect demonstrated a significant overall positive effect of experimental interventions on cognition (*g* = 0.55; 95% CI = 0.22, 0.89; *p* = 0.0012) (Fig. 3 and Table 5). Heterogeneity was significant (*Q*(14) = 36.12, *p* = 0.001; attention: *τ*^2^ = 0.19; *I*^2^ = 56.6%; executive functions: *τ*^2^ = 0.15; *I*^2^ = 52.8%; working memory: *τ*^2^ = 0.38; *I*^2^ = 59.1%; immediate memory: *τ*^2^ = 0.00; *I*^2^ = 0.0%; language: *τ*^2^ = 0.00; *I*^2^ = 0.0%). Two studies were identified as potential outliers, with absolute studentized deleted residuals greater than 1.96: Fuentes & Kerr (2017) (*z* = 2.51 for working memory and *z* = 2.27 for attention) and Khaleghi et al. (2024) (*z* = 2.07). Cook’s distance values were highest for Fuentes & Kerr (2017) (*D* = 0.39) and Khaleghi et al. (2024) (*D* = 0.18), indicating that these studies might be influential in the model. However, DFBETAs did not exceed the threshold of ± 1, suggesting no single study had a disproportionate impact on the overall effect size estimate. As the studies by Fuentes & Kerr (2017) and Khaleghi et al. (2024) were identified both as potential outliers and influential cases, the meta-analysis was run again without them. The heterogeneity decreased (*Q*(11) = 17.06, *p* = 0.11; attention: *τ*^2^ = 0.03; *I*^2^ = 25.0%; executive functions: *τ*^2^ = 0.07; *I*^2^ = 32.3%; working memory: *τ*^2^ = 0.16; *I*^2^ = 47.2%; immediate memory: *τ*^2^ = 0.00; *I*^2^ = 0.0%; language: *τ*^2^ = 0.00; *I*^2^ = 0.0%). Given the consistent positive effect, all studies were retained in all subsequent analyses to maximize statistical power and maintain ecological validity, and the influence of moderator variables was examined. Egger’s regression intercept suggested potential small-study effects (Intercept = 1.83; SE = 0.38; 95% CI = 1.10, 2.57; *z* = 4.88; *p* < 0.0001). The adjusted effect size under the three-parameter selection model (*g* = 0.86; 95% CI = 0.11, 1.60; *p* = 0.02) did not significantly differ from the unadjusted effect size (LRT = 1.42, *p* = 0.23).

**Table 5:** Effect sizes of multivariate meta-analysis of pre-post changes in experimental interventions in pediatric patients

Effect size by outcome	*k*	Hedges’ *g*		95% CI		95% prediction intervals	
		*g*	*p*	Lower	Upper	Lower	Upper
Overall effect on cognition	7	0.55	0.0012	0.22	0.89	−0.70	1.81
Cognitive outcome							
Attention	6	0.66	0.01	0.15	1.17	−0.89	2.20
Executive functions	3	0.43	0.09	−0.07	0.94	−0.68	1.55
Working memory	4	0.80	0.06	−0.05	1.65	−1.58	3.19
Immediate episodic memory	1	0.65	0.16	−0.25	1.56	−0.53	1.84
Language	1	0.39	0.26	−0.29	1.06	−0.50	1.27

Regarding the moderating role of the cognitive outcome, the results revealed a trend of variability in effect sizes across cognitive domains (QM(5) = 10.16, *p* = 0.07). Heterogeneity was significant (*Q*(10) = 34.59, *p* < 0.0001; attention: *τ*^2^ = 0.29; *I*^2^ = 66.8%; executive functions: *τ*^2^ = 0.12; *I*^2^ = 46.9%; working memory: *τ*^2^ = 0.67; *I*^2^ = 71.7%; immediate memory: *τ*^2^ = 0.00; *I*^2^ = 0.0%; language: *τ*^2^ = 0.00; *I*^2^ = 0.0%). The effects of experimental interventions were significant for attention (*g* = 0.66; 95% CI = 0.15, 1.17; *p* = 0.01) and tended to be significant for working memory (*g* = 0.80; 95% CI = −0.05, 1.65; p = 0.06), but not for executive function (*g* = 0.43; 95% CI = −0.07, 0.94; *p* = 0.09), immediate episodic memory (*g* = 0.65; 95% CI = −0.25, 1.56; *p* = 0.16), or language (*g* = 0.39; 95% CI = −0.29, 1.06; *p* = 0.26). Any study examined delayed episodic memory. The prediction intervals for each outcome are displayed in Table 5.

The multivariate meta-regression model examining the influence of study-level moderators revealed no significant overall moderation effect (QM(7) = 3.89, *p* = 0.79). Heterogeneity was significant (*Q*(7) = 31.23, *p* < 0.0001; attention: *τ*^2^ = 0.30; *I*^2^ = 67.1%; executive functions: *τ*^2^ = 0.61; *I*^2^ = 81.7%; working memory: *τ*^2^ = 0.90; *I*^2^ = 77.2%; immediate memory: *τ*^2^ = 0.00; *I*^2^ = 0.0%; language: *τ*^2^ = 0.00; *I*^2^ = 0.0%). No significant differences were found depending on the use of compensatory strategies (*B* = 0.42; SE = 0.73; 95% CI = −1.01, 1.85; *p* = 0.56), the intervention duration (*B* = −0.50; SE = 0.37; 95% CI = −1.23, 0.23; *p* = 0.18), and the level of methodological evidence (*B* = 0.72; SE = 0.43; 95% CI = −0.12, 1.55; *p* = 0.09). All experimental interventions included non-surgical patients, used brain training games, and had an individual format, so no subgroup analyses were carried out depending on these variables.

In the above analyses, a constant correlation of 0.5 between effect sizes within the same study was assumed (Cheung, 2019). Sensitivity analyses using 0 and 0.8 correlation values yielded comparable results. The only notable difference was observed when a correlation of 0.8 was applied: the effect of the experimental interventions on executive function, previously non-significant (g = 0.43; 95% CI = −0.07, 0.94; *p* = 0.09), reached statistical significance (g = 0.58; 95% CI = 0.07, 1.08; *p* = 0.026). All other outcomes remained consistent across the different correlation assumptions, supporting the robustness of the overall findings.

### Meta-analysis Comparing Experimental Interventions and Control Groups of Patients with Epilepsy Who Did Not Receive Neuropsychological Intervention

#### Studies with Adult Patients

**Fig. 4:**
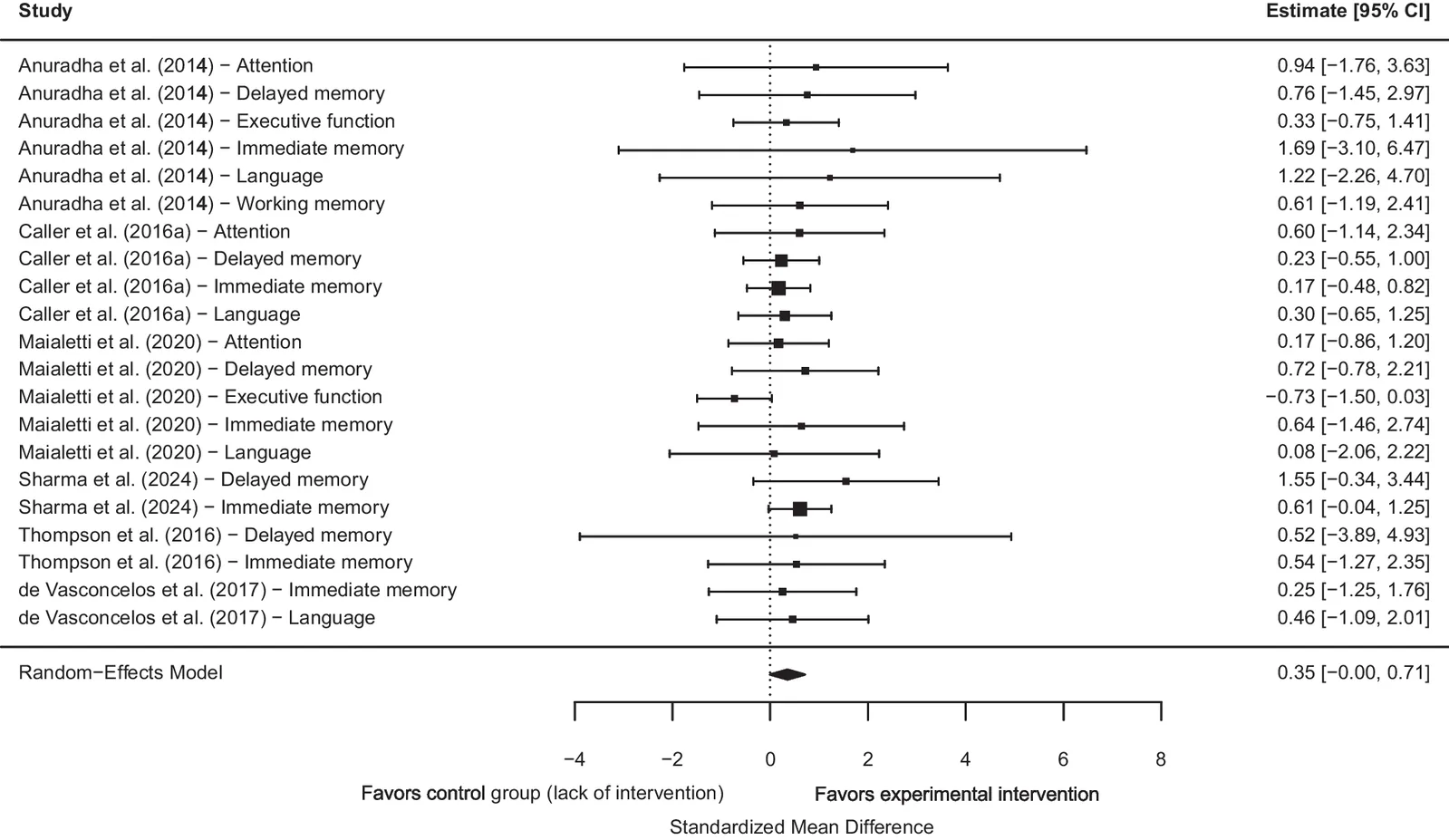
Forest plot comparing the effectiveness of neuropsychological interventions versus control groups of patients without neuropsychological intervention in improving cognitive function in adult samples (overall effect)

Six studies were included in the multivariate meta-analysis comparing experimental interventions and control groups of patients with epilepsy who did not receive neuropsychological intervention in adult samples (229 participants: 136 participants in experimental interventions and 93 participants in control groups). We found an overall positive effect of the experimental intervention on cognition, although the effect did not reach statistical significance (*g* = 0.35; 95% CI = −0.00, 0.71; *p* = 0.053) (Fig. 4 and Table 6). Heterogeneity was not significant (*Q*(20) = 20.32, *p* = 0.44; attention: *τ*^2^ = 0.05; *I*^2^ = 4.7%; executive functions: *τ*^2^ = 0.70; *I*^2^ = 48.4%; working memory: *τ*^2^ = 0.00; *I*^2^ = 0.0%; immediate memory: *τ*^2^ = 0.00; *I*^2^ = 0.2%; delayed memory: *τ*^2^ = 0.00; *I*^2^ = 0.0%; and language: *τ*^2^ = 0.04; *I*^2^ = 3.5%). The study by Maialetti et al. (2020) was identified as a potential outlier (*z* = 2.50). Cook’s distance identified two studies as having potentially higher influence on the model: Caller et al. (2016a) (*D* = 0.48) and Thompson et al. (2016) (*D* = 0.41). However, DFBETAs did not exceed the threshold of ± 1, suggesting no single study had a disproportionate impact on the overall effect size estimate. No study was identified as an influential case across all diagnostics, so all studies were retained in the subsequent analyses, and the influence of potential moderators was examined. Egger’s regression intercept suggested potential small-study effects (Intercept = 0.83; SE = 0.34; 95% CI = 0.16, 1.50; *z* = 2.42; *p* = 0.0155). The adjusted effect size under the three-parameter selection model (*g* = 0.57; 95% CI = −0.13, 1.27; *p* = 0.11) did not significantly differ from the unadjusted estimate (LRT = 2.11, *p* = 0.15).

**Table 6:** Effect sizes of multivariate meta-analysis comparing experimental interventions and control groups of patients with epilepsy without neuropsychological intervention in adult patients

Effect size by outcome	*k*	Hedges’ *g*		95% CI		95% prediction intervals	
		*g*	*p*	Lower	Upper	Lower	Upper
Overall effect on cognition	6	0.35	0.053	−0.00	0.71	−0.59	1.30
Cognitive outcome							
Attention	3	0.43	0.31	−0.41	1.28	−0.93	1.80
Executive functions	2	−0.33	0.49	−1.27	0.60	−2.47	1.81
Working memory	1	0.27	0.71	−1.14	1.68	−1.73	2.26
Immediate episodic memory	6	0.38	0.066	−0.02	0.79	−0.26	1.02
Delayed episodic memory	5	0.57	0.10	−0.10	1.24	−0.67	1.81
Language	4	0.30	0.39	−0.39	0.99	−0.75	1.35

When the cognitive outcome was included as a moderator, the results showed no significant variability in effect sizes across cognitive domains (QM(6) = 5.84, *p* = 0.44). Heterogeneity was not significant (*Q*(15) = 8.35, *p* = 0.91; attention: *τ*^2^ = 0.06; *I*^2^ = 5.6%; executive functions: *τ*^2^ = 0.36; *I*^2^ = 32.7%; working memory: *τ*^2^ = 0.00; *I*^2^ = 0.0%; immediate memory: *τ*^2^ = 0.01; *I*^2^ = 0.7%; delayed memory: *τ*^2^ = 0.08; *I*^2^ = 4.7%; and language: *τ*^2^ = 0.02; *I*^2^ = 1.9%). The positive effect of the experimental interventions versus the control interventions tended to be significant for immediate episodic memory (*g* = 0.38; 95% CI = −0.02, 0.79; *p* = 0.066). However, the effects were not significant for attention (*g* = 0.43; 95% CI = −0.41, 1.28; *p* = 0.31), executive function (*g* = −0.33; 95% CI = −1.27, 0.60; *p* = 0.49), working memory (*g* = 0.27; 95% CI = −1.14, 1.68; *p* = 0.71), delayed episodic memory (*g* = 0.57; 95% CI = −0.10, 1.24; *p* = 0.10), and language (*g* = 0.30; 95% CI = −0.39, 0.99; *p* = 0.39). The number of studies for each outcome, the effect sizes, and the prediction intervals are shown in Table 6.

The multivariate meta-regression model examining the influence of study-level moderators showed a non-significant overall moderation effect (QM(8) = 13.74, *p* = 0.09). Heterogeneity was not significant (*Q*(12) = 3.23, *p* = 0.99; attention: *τ*^2^ = 0.01; *I*^2^ = 1.1%; executive functions: *τ*^2^ = 0.03; *I*^2^ = 3.3%; working memory: *τ*^2^ = 0.00; *I*^2^ = 0.0%; immediate memory: *τ*^2^ = 0.00; *I*^2^ = 0.0%; delayed memory: *τ*^2^ = 0.00; and *I*^2^ = 0.1%; language: *τ*^2^ = 0.00; *I*^2^ = 0.5%). The use of brain training games was associated with a decrease in cognitive functioning (*B* = −1.03; SE = 0.52; 95% CI = −2.05, −0.00; *p* = 0.049). No significant differences were found depending on the intervention duration (*B* = 0.43; SE = 0.29; 95% CI = −0.14, 1.01; *p* = 0.14) and the level of methodological evidence (*B* = −0.66; SE = 0.46; 95% CI = −1.56, 0.23; *p* = 0.14). Only one study included non-surgical patients and exclusively internal compensatory strategies (not external), and the intervention format was individual in all studies, so no subgroup analyses were carried out depending on these variables.

In the above analyses, a constant correlation of 0.5 between effect sizes within the same study was assumed (Cheung, 2019). Sensitivity analyses using a correlation value of 0 showed that the overall effect of experimental interventions versus control groups on cognition changed from a trend to a significant effect (*g* = 0.39; 95% CI = 0.10, 0.68; *p* = 0.008). In contrast, assuming a correlation of 0.8 rendered the effect non-significant (g = 0.22; 95% CI = −0.13, 0.57; *p* = 0.21), increased heterogeneity to a significant level (*Q*(20) = 47.79, *p* < 0.0001; *τ*^2^ = 0.17; *I*^2^ = 58.2%), and attenuated the moderator effect of brain training games from significant to a trend (*B* = −0.86; SE = 0.48; 95% CI = −1.80, 0.08; *p* = 0.07). This indicates that the overall effect and some moderator effects may be sensitive to the assumed within-study correlation; however, the direction of effects remained consistent across models.

### Studies with Pediatric Patients

**Fig. 5:**
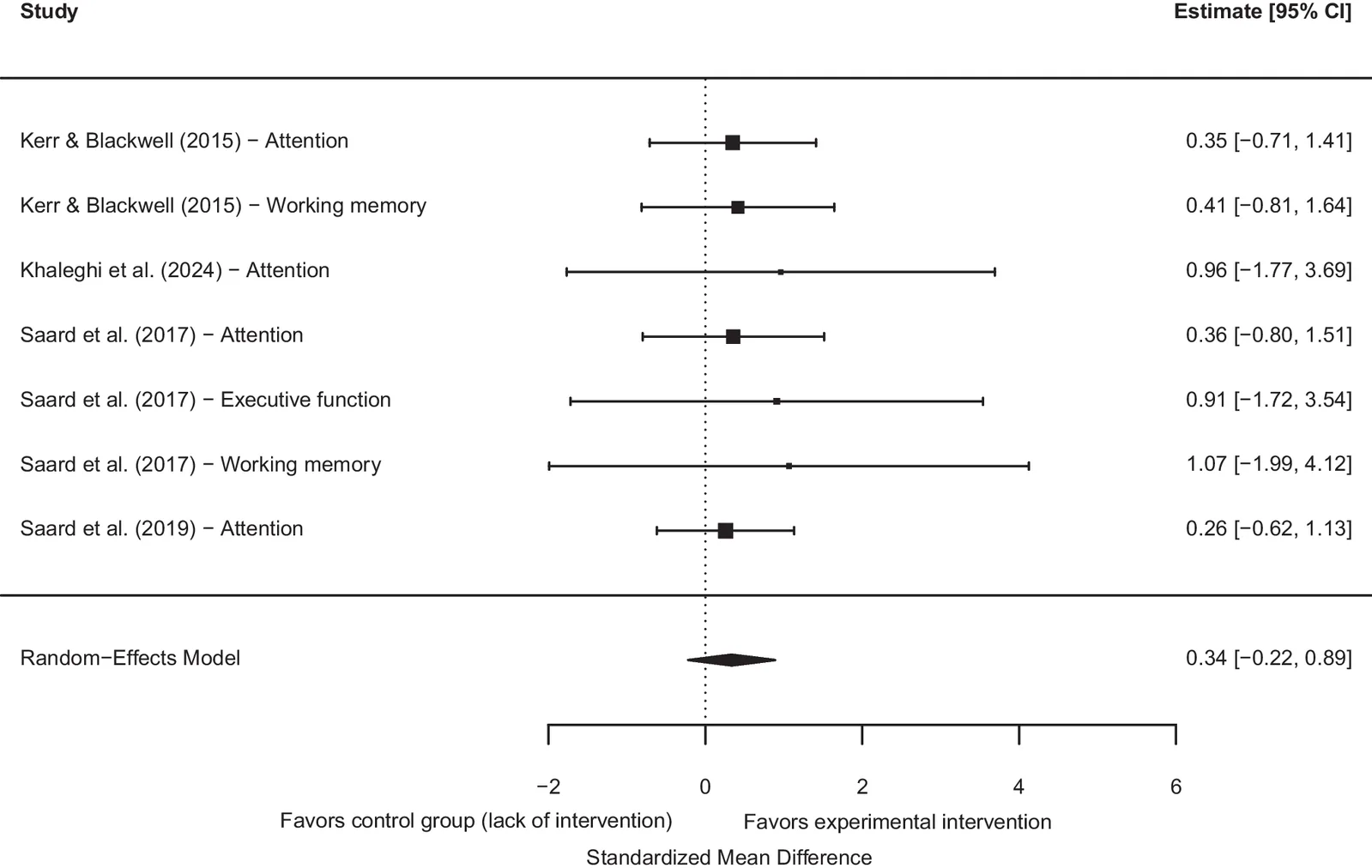
Forest plot comparing the effectiveness of neuropsychological interventions versus control groups of patients without neuropsychological intervention in improving cognitive function in pediatric samples (overall effect)

Four studies were included in the multivariate meta-analysis comparing experimental interventions and control groups of patients with epilepsy who did not receive neuropsychological intervention in pediatric samples (171 participants: 101 participants in experimental interventions and 70 participants in control groups). The overall effect of the experimental intervention on cognition was not significant (*g* = 0.34; 95% CI = −0.22, 0.89; *p* = 0.24) (Fig. 5 and Table 7). Heterogeneity was not significant (*Q*(6) = 0.62, *p* = 0.99; attention: *τ*^2^ = 0.00; *I*^2^ = 0.0%; executive functions: *τ*^2^ = 0.00; *I*^2^ = 0.0%; working memory: *τ*^2^ = 0.00; *I*^2^ = 0.0%). No studies were identified as potential outliers through studentized deleted residuals. Cook’s distance values were low for all studies (maximum *D* = 0.03 for Saard et al., 2019), and DFBETAs did not exceed the threshold of ± 1, suggesting no single study had a disproportionate impact on the overall effect size estimate. Egger’s regression intercept suggested no significant small-study effects (Intercept = 1.20; SE = 1.23; 95% CI = −1.20, 3.61; *z* = 1.00; *p* = 0.33). The adjusted effect size under the three-parameter selection model (*g* = 0.67; 95% CI = −0.23, 1.56; *p* = 0.14) did not significantly differ from the unadjusted estimate (LRT = 1.71, *p* = 0.19).

**Table 7:** Effect sizes of multivariate meta-analysis comparing experimental interventions and control groups of patients with epilepsy without neuropsychological intervention in pediatric patients

Effect size by outcome	*k*	Hedges’ *g*		95% CI		95% prediction intervals	
		*g*	*p*	Lower	Upper	Lower	Upper
Overall effect on cognition	4	0.34	0.24	−0.22	0.89	−0.88	1.55
Cognitive outcome							
Attention	4	0.32	0.27	−0.24	0.88	−0.92	1.56
Executive functions	1	0.72	0.53	−1.51	2.95	−4.18	5.61
Working memory	2	0.48	0.37	−0.58	1.54	−1.84	2.81

When the cognitive outcome was included as a moderator, the results showed no significant variability in effect sizes across cognitive domains (QM(3) = 1.61, *p* = 0.66). It is worth noting that this meta-analysis only evaluated attention, executive function, and working memory. Heterogeneity was not significant (*Q*(4) = 0.42, *p* = 0.98; attention: *τ*^2^ = 0.00; *I*^2^ = 0.0%; executive functions: *τ*^2^ = 0.00; *I*^2^ = 0.0%; and working memory: *τ*^2^ = 0.00; *I*^2^ = 0.0%). The effects were not significant for attention (*g* = 0.32; 95% CI = −0.24, 0.88; *p* = 0.27), executive function (*g* = 0.72; 95% CI = −1.51, 2.95; *p* = 0.53), or working memory (*g* = 0.48; 95% CI = −0.58, 1.54; *p* = 0.37). The number of studies for each outcome, the effect sizes, and the prediction intervals are shown in Table 7.

The multivariate meta-regression model examining the influence of study-level moderators revealed no significant overall moderation effect (QM(4) = 0.43, *p* = 0.98). Heterogeneity was not significant (*Q*(2) = 0.20, *p* = 0.91; attention: *τ*^2^ = 0.00; *I*^2^ = 0.0%; executive functions: *τ*^2^ = 0.00; *I*^2^ = 0.0%; working memory: *τ*^2^ = 0.00; *I*^2^ = 0.0%). No significant differences were found depending on the intervention duration (*B* = −1.25; SE = 2.91; 95% CI = −6.95, 4.45; *p* = 0.67) and the level of methodological evidence (*B* = 2.04; SE = 4.53; 95% CI = −6.84, 10.93; *p* = 0.65). All interventions included non-surgical patients, utilized brain training games, employed internal compensatory strategies, and had an individual format; therefore, no subgroup analyses were conducted based on these variables.

In the above analyses, a constant within-study correlation of 0.5 was assumed (Cheung, 2019). Sensitivity analyses using 0 and 0.8 correlation values yielded similar results, suggesting that the findings were robust to different correlation assumptions.

## Discussion

This meta-analysis evaluated the effectiveness of neuropsychological interventions in enhancing cognitive functioning in patients with epilepsy. Twenty-five studies that met the inclusion criteria were selected. The results show that neuropsychological interventions significantly improved overall cognition for both adult and pediatric patients, with a moderate effect size. Furthermore, comparisons between neuropsychological interventions and control groups of patients with epilepsy who did not receive neuropsychological intervention (e.g., waiting list) revealed a trend toward a positive overall effect of neuropsychological interventions on cognition in adults, while no significant effects were observed for pediatric patients.

In studies involving adult patients, the meta-analysis of pre-post changes in neuropsychological interventions showed significant improvements in attention, immediate and delayed episodic memory, and language, with moderate effect sizes. Notably, all interventions incorporated targeted memory techniques (e.g., imagery mnemonics, method of loci, and semantic elaboration), and most of them used specific attention tasks, which likely contributed to the moderate effect sizes observed in these outcomes. Attention is a core cognitive domain often affected in epilepsy (Auvin et al., 2018; Cano-López et al., 2025) and plays a crucial role in supporting higher-order functions such as memory (Stuss & Alexander, 2000). Given that memory deficits are among the main concerns of patients with epilepsy (Thompson et al., 2016), and that many studies included samples with TLE—in which memory impairment constitutes the core cognitive deficit (Lin et al., 2012)—memory-focused interventions may have been particularly well suited to these cognitive profiles, thereby amplifying the intervention effects. Interestingly, improvements in language performance were observed even though only three out of the eight neuropsychological interventions examining this outcome incorporated explicit language-focused strategies (de Vasconcelos et al., 2017; Maschio et al., 2015; Schwarz et al., 2022). Language outcomes mainly included naming by visual confrontation, which implies access to visual storage and the subsequent assignation of a word or semantic load, and semantic fluency tasks that require associative processes between elements of a category (Cano-López et al., 2017). These findings suggest that language may benefit indirectly from broader neuropsychological interventions: enhanced attentional engagement and mnemonic strategies could support lexical access and semantic organization, improving language performance even without direct language rehabilitation. This interpretation is supported by neuroimaging studies showing that semantic retrieval relies on distributed networks involving attentional and mnemonic processes, beyond classical language areas (Geranmayeh et al., 2014).

Despite the observed benefits in attention, episodic memory, and language, neuropsychological interventions did not yield significant effects on executive functions or working memory in adult patients. This may be explained by several factors. First, these domains were less explicitly targeted in most interventions, and evidence suggests that improving them requires intensive practice of complex tasks involving planning, cognitive flexibility, and working memory (Diamond, 2013). Second, these functions are particularly vulnerable to the cognitive side effects of ASMs (Hessen et al., 2009; Witt et al., 2015), which may impair neuroplasticity and limit patients’ capacity to benefit from cognitive interventions. Third, although the tasks used to assess working memory were relatively homogeneous, the measures of executive function showed greater variability, which may have limited the comparability across studies and reduced the likelihood of detecting consistent effects. These findings highlight the need for more focused and intensive interventions tailored to patients’ cognitive profiles, particularly to enhance executive functions and working memory. Given that attention, executive functions, and working memory have been shown to differentiate episodic memory profiles in epilepsy patients (Lozano-García et al., 2024b) and are key contributors to daily functioning (Catalán-Aguilar et al., 2022; Stretton & Thompson, 2012) and quality of life (Lozano-García et al., 2021), targeting these domains may be essential for optimizing neuropsychological outcomes in this population.

In pediatric samples, the meta-analysis of pre-post changes in neuropsychological interventions revealed significant improvements in attention (moderate effect size) and a trend toward a large positive effect on working memory. No significant effects were observed for executive function, episodic memory, or language. These results may be explained by the fact that most interventions focused on attention (Glynn et al., 2016; Khaleghi et al., 2024; Saard et al., 2017, 2019) and working memory (Fuentes & Kerr, 2017; Glynn et al., 2016; Kerr & Blackwell, 2015; Khaleghi et al., 2024), with only one study addressing inhibitory control (Tapia et al., 2024). Unlike in adult samples, none of the interventions directly targeted episodic memory. Additionally, attention and working memory were the cognitive domains most frequently assessed, with attention being evaluated in six studies and working memory being assessed in four studies. Nevertheless, executive function was evaluated in three studies, and only one study evaluated immediate episodic memory and language. It has been suggested that meta-analyses based on fewer than five outcomes should be interpreted with caution due to limited statistical power (Jackson & Turner, 2017), which may partially explain the absence of significant effects in these domains. Nonetheless, because working memory supports encoding, retrieval, and resistance to interference (Stuss & Alexander, 2000), interventions focused on this domain may promote broader cognitive improvements. Future research should evaluate neuropsychological interventions more comprehensively in pediatric epilepsy, particularly including episodic memory outcomes.

When comparing the effectiveness of neuropsychological interventions to control groups of patients with epilepsy who did not receive such interventions, a trend toward a positive moderate effect on overall cognition was observed in adult patients, while no significant effects were found in pediatric samples. These findings should be interpreted with caution due to the limited number of studies included in the meta-analyses, particularly in the pediatric group, where only four studies were available for analysis, and only attention, executive function, and working memory were evaluated. In adults, when examining cognitive domains individually, the positive effect observed tended to be significant for immediate episodic memory, a domain assessed in all six studies included in this meta-analysis. No significant effects were found in other domains, but these were each evaluated in fewer than five studies (except delayed memory), limiting interpretability. Importantly, all but one of the adult studies included surgical patients. Although the meta-analysis examining the effectiveness of neuropsychological interventions (i.e., without comparison with control groups) did not detect significant differences based on epilepsy treatment, neurosurgical procedures may lead to widespread brain network changes (Foesleitner et al., 2020; Sainburg et al., 2025) and are associated with cognitive deficits (Sherman et al., 2011), limiting the potential for recovery. Even so, Mazur-Mosiewicz et al. (2015) found that post-surgical patients benefited from memory-focused interventions, though attention improvements were more likely attributable to surgical effects. Further studies are needed to evaluate the effectiveness of neuropsychological interventions in comparison with control groups in non-surgical patients, to better isolate intervention effects from postsurgical recovery trajectories.

Regarding intervention characteristics, the use of brain training games was associated with decreased cognitive functioning in the meta-analysis comparing the effectiveness of neuropsychological interventions versus control groups in adult samples. In pediatric samples, all studies included in the meta-analyses employed brain training games, which prevented analysis of the potential influence of this factor. These findings align with a recent systematic review in healthy older adults and individuals with mild cognitive impairment, which concluded that, while commercial brain training games may have recreational value, there is limited empirical support for their effectiveness in improving general cognition, memory, or daily functioning (Nguyen et al., 2022). The negative association observed in our analysis may therefore reflect both the limited cognitive benefits of these programs and the inflated expectations generated by their commercial marketing—an issue previously highlighted in a consensus report by the Max Planck Institute for Human Development and the Stanford Center on Longevity (2014).

No differences in the effectiveness of the intervention were found depending on the type of compensatory strategies used. Accordingly, both internal and external compensatory strategies have been shown to improve daily functioning in individuals with memory impairment due to acquired brain injury (Gopi et al., 2022). Evidence suggests that internal strategies may be more suitable for individuals with mild impairments, while external aids could be more beneficial for those with more severe deficits (Cicerone et al., 2019). However, even when these strategies are effective in structured rehabilitation contexts, patients often struggle to implement them independently in everyday situations (Wilson, 2000, 2002).

The format and the duration of the intervention were not significant modulators of the effectiveness of the neuropsychological interventions. Intervention format could only be examined in the meta-analysis focused on pre-post changes in cognition in neuropsychological interventions with adults, and only two studies employed an exclusively group-based format. Current evidence in patients with acquired brain injury supports the use of group-based strategies to improve memory, executive function, and language (Cicerone et al., 2019). However, given the limited number of group-based interventions included in our analyses, further research is needed to clarify their relative effectiveness in patients with epilepsy. Intervention duration varied considerably in length, from 6 (Icellioglu et al., 2017) to 42 h (Schwarz et al., 2022) in adult studies and from 3.13 (Fuentes & Kerr, 2017; Kerr & Blackwell, 2015) to 9 h (Khaleghi et al., 2024) in pediatric studies. These durations slightly differ from those reported in the systematic review, as they refer only to the subset of interventions that could be included in the quantitative analyses. Although previous studies in other populations have associated interventions of at least 15 h with greater effectiveness (Gómez-Cáceres et al., 2023), the absence of significant differences according to intervention characteristics in our meta-analysis could be explained by the marked heterogeneity in intervention protocols and outcome measures. This variability highlights that, beyond demographic and clinical factors, the effectiveness of cognitive rehabilitation may depend more on the use of tailored and patient-specific strategies that align with each individual’s cognitive profile. Interestingly, higher methodological quality was significantly associated with greater intervention effectiveness in adult studies, indicating a potential influence of study rigor on observed outcomes.

This study has the following strengths. First, to our knowledge, it is the first meta-analysis to examine the effectiveness of neuropsychological interventions in enhancing cognitive functioning in patients with epilepsy. Second, we analyzed pre-post changes in cognitive outcomes following neuropsychological intervention, as well as whether these changes differed from those observed in control groups of patients with epilepsy who did not receive neuropsychological intervention. Third, the meta-analyses examined the effectiveness of neuropsychological interventions in both adult and pediatric samples, as recommended (Joplin et al., 2018). Finally, we explored potential moderators such as epilepsy treatment, intervention duration, type, format, and methodological quality.

Despite these strengths, this meta-analysis has limitations. First, the sample of the reviewed studies was heterogeneous in terms of epilepsy type and seizure control. Second, although pre-post changes were analyzed for all cognitive outcomes after neuropsychological intervention, a limited number of studies included a control group of patients with epilepsy, and this reduces the statistical power for comparisons. Third, results from meta-analyses based on fewer than five outcomes should be interpreted with caution due to limited statistical power (Jackson & Turner, 2017) and potentially unstable estimates, as reflected in the width of the confidence intervals. Fourth, heterogeneity was significant in the meta-analysis examining the effectiveness of neuropsychological interventions in improving cognitive function in pediatric patients. Despite the effects remaining significant when outliers were excluded, results should be interpreted with caution. Fifth, Egger’s test indicated potential small-study effects in most meta-analyses; however, the differences between adjusted and unadjusted estimates in the three-parameter selection model did not reach statistical significance. Sixth, all pediatric studies involved non-surgical patients, used brain training games, and followed an individual format; thus, future research should examine the influence of surgical status, alternative intervention types, and different delivery formats in this age group. Finally, most studies did not include follow-ups after the end of the intervention, so conclusions about long-term effectiveness cannot be drawn.

Cognitive impairment is one of the most prevalent comorbidities in epilepsy (Fisher et al., 2014) that may be aggravated by antiepileptic treatments (Cano-López et al., 2017; Lozano-García et al., 2024a). This meta-analysis shows that neuropsychological interventions are effective in improving cognitive function in adults and pediatric samples with epilepsy. However, further controlled studies are needed to compare these interventions with no treatment, as the evidence remains limited. Considering the findings of the reviewed studies, it is possible to provide certain recommendations for future studies. First, cognitive improvement was generally defined by statistically significant pre-post changes, with only four studies also assessing clinically meaningful change (de Vasconcelos et al., 2017; Fuentes & Kerr, 2017; Kerr & Blackwell, 2015; Thompson et al., 2016). Although effect sizes represent the magnitude of change at the group level and do not inherently indicate clinical significance (Davis et al., 2021), in populations with prominent cognitive complaints—such as individuals with epilepsy—the moderate effect sizes observed may reflect clinically meaningful improvements in neuropsychological function. Nevertheless, given the importance of establishing clinical relevance in neuropsychological outcomes (Duff, 2012), future studies should incorporate measures such as reliable change indices. Second, as stated by Baxendale (2020), cognitive rehabilitation is inherently time- and resource-intensive, and the current scarcity of experimental studies in patients with epilepsy likely reflects the complexity of this intervention and a lack of funding for cognitive care in this population. Future efforts should focus on conducting randomized controlled trials with larger samples to refine cognitive rehabilitation protocols, define patient profiles and clinical contexts for their application, and support integration into routine care for people with epilepsy. Third, there is limited information in the reviewed studies on specific memory processes affected in each case (encoding, consolidation, and retrieval) and how interventions are tailored to these individual profiles. Future research should address this gap by clearly characterizing memory deficits and designing targeted strategies accordingly. Finally, it is essential to determine whether the abilities acquired through intervention transfer to daily life and are sustained over time. Future studies should therefore include measures of functional independence and incorporate multiple post-intervention follow-up assessments.

## Supplementary Information

Below is the link to the electronic supplementary material.

ESM1 (DOCX 22.4 KB)

## Data Availability

This review is registered in PROSPERO (CRD42024625503). Data files and the code used for the meta-analyses are available at the following link: https://osf.io/gake7/?view_only=f6b50c4b35ea40b782435192dc9facc5.
