# Supplementary material for: Examining the Effectiveness of Neuropsychological Interventions on Cognition in Patients with Epilepsy: A Systematic Review and Meta-Analysis
Source: Neuropsychol Rev. 2025 Oct 23;36(3):388–427. doi: 10.1007/s11065-025-09678-2 (PMC13653837; doi:10.1007/s11065-025-09678-2)
Supplement: Supplementary file 1 [file 11065_2025_9678_MOESM1_ESM.docx]

**Supplementary material**

1. **Computation of effect sizes (Hedges’ g) and sampling variance**
   1.1. **Meta-analyses of pre-post changes in experimental interventions**

For meta-analyses examining the pre-post changes in experimental interventions, *g* was computed as the mean difference between post- and pre-intervention scores divided by the standard deviation of the pretest scores (Morris & DeShon, 2002).

The formula used was:

*g* = $\left( \frac{M_{post}- M_{pre}}{{SD}_{pre}} \right)\cdot J$

Where:

- M_pre_ and M_post_ are the pre- and post-intervention means,
- SDpre is the standard deviation at pretest,
- J = 1 − (3 / (4n − 1)) is the correction for small sample bias.

The sampling variance of *g* was calculated as:

$$Var (g) = \left[ \left( \frac{2 (1- r)}{n} \right)\cdot\left( \frac{n - 1}{n - 3} \right)\cdot\left( 1 + \frac{n d^{2}}{2 \left( 1-r \right)} \right)- d^{2} \right]\cdot J^{2}$$

Where:

- d is the uncorrected effect size,
- r is the estimated pre-post correlation,
- n is the sample size of the group.
  1.2. **Meta-analyses comparing experimental and control groups of patients with epilepsy without neuropsychological intervention**

For meta-analyses comparing the pre-post changes between experimental and control groups of patients with epilepsy without neuropsychological intervention, effect sizes were computed as the difference between the standardized mean changes of the experimental and control interventions, divided by a pooled estimate of the pretest standard deviation (Morris, 2008).

The formula used was:

$${dg}^{3}= c(m) \left[ \frac{\left( \bar{Y_{post}^{T}}- \bar{Y_{pre}^{T}} \right)- \left( \bar{Y_{post}^{C}}- \bar{Y_{pre}^{C}} \right)}{\bar{S_{pre}}} \right]$$

Where:

- $\bar{Y}_{pre}^{T}$ and $\bar{Y}_{post}^{T}$are the pre- and post-test means for the treatment group,
- $\bar{Y}_{pre}^{C}$ and $\bar{Y}_{post}^{C}$ are the pre- and post-test means for the control group,
- $\bar{S}_{pre}$ is the pooled standard deviation at pretest, calculated as:

$$S_{pre}=\sqrt{\frac{\left( n_{T} - 1 \right)\left( S_{pre}^{T} \right)^{2} + \left( n_{C} - 1 \right)\left( S_{pre}^{C} \right)^{2}}{n_{T} + n_{C} - 2}}$$

- Where:
  - $n_{T}$: sample size of the treatment group.
  - $n_{C}$: sample size of the control group.
  - $\left( S_{pre}^{T} \right)^{2}$: pretest variance of the treatment group.
  - $\left( S_{pre}^{C} \right)^{2}:$ pretest variance of the control group.
- $c(m) = 1-\frac{3}{4\left( n_{T} + n_{C} \right) - 9}$ is the correction factor for small sample bias.

2. **Prediction intervals computation**

This section describes the formula used to compute 95% prediction intervals in the meta-analyses, according to the approach proposed by Borenstein (2024).

The formula used was:

PI = M$\pm t_{df}^{\propto} \sqrt{T^{2}+V_{M}}$

Where:

- M is the estimated mean effect size,
- T² (tau-squared) is the estimated between-study variance,
- V_M_ is the error variance of the mean,
- *t* is the critical value from the t-distribution with df = k − 2 degrees of freedom (k = number of studies).

**References**

Borenstein M. (2024). Avoiding common mistakes in meta-analysis: Understanding the distinct roles of Q, I-squared, tau-squared, and the prediction interval in reporting heterogeneity. *Research Synthesis Methods, 15*(2), 354-368. <https://doi.org/10.1002/jrsm.1678>

Morris, S. B. (2008). Estimating effect sizes from pretest-posttest-control group designs. *Organizational Research Methods, 11*(2), 364-386. <https://doi.org/10.1177/1094428106291059>

Morris, S. B., & DeShon, R. P. (2002). Combining effect size estimates in meta-analysis with repeated measures and independent-groups designs. *Psychological Methods, 7*(1), 105-125. <https://doi.org/10.1037/1082-989x.7.1.105>
